# Constructing Donor–Acceptor-Linked COFs Electrolytes to Regulate Electron Density and Accelerate the Li^+^ Migration in Quasi-Solid-State Battery

**DOI:** 10.1007/s40820-024-01509-y

**Published:** 2024-09-26

**Authors:** Genfu Zhao, Hang Ma, Conghui Zhang, Yongxin Yang, Shuyuan Yu, Haiye Zhu, Yongjiang Sun, Hong Guo

**Affiliations:** 1 School of Materials and Energy, International Joint Research Center for Advanced Energy Materials of Yunnan Province, Yunnan University, Kunming, 650091 People’s Republic of China; 2 Yunnan Yuntianhua Co., Ltd, R & D Center, Kunming, 650228 People’s Republic of China; 3 Southwest United Graduate School, Kunming, 650091 People’s Republic of China

**Keywords:** Electronic modulation engineering, Donor–acceptor-linked covalent organic frameworks, Quasi-solid-state Li metal battery

## Abstract

Regulation the electronic density of solid-state electrolyte by donor–acceptor (D–A) system can achieve highly-selective Li^+^ transportation and conduction in solid-state Li metal batteries. This study reports a high-performance solid-state electrolyte thorough D–A-linked covalent organic frameworks (COFs) based on intramolecular charge transfer interactions. Unlike other reported COF-based solid-state electrolyte, the developed concept with D–A-linked COFs not only achieves electronic modulation to promote highly-selective Li^+^ migration and inhibit Li dendrite, but also offers a crucial opportunity to understand the role of electronic density in solid-state Li metal batteries. The introduced strong electronegativity F-based ligand in COF electrolyte results in highly-selective Li^+^ (transference number 0.83), high ionic conductivity (6.7 × 10^–4^ S cm^−1^), excellent cyclic ability (1000 h) in Li metal symmetric cell and high-capacity retention in Li/LiFePO_4_ cell (90.8% for 300 cycles at 5C) than substituted C- and N-based ligands. This is ascribed to outstanding D–A interaction between donor porphyrin and acceptor F atoms, which effectively expedites electron transferring from porphyrin to F-based ligand and enhances Li^+^ kinetics. Consequently, we anticipate that this work creates insight into the strategy for accelerating Li^+^ conduction in high-performance solid-state Li metal batteries through D–A system.

**Supplementary Information:**

The online version contains supplementary material available at 10.1007/s40820-024-01509-y.

## Highlights

- Donor–acceptor-linked covalent organic framework (COF)-based electrolyte can not only fulfill highly-selective Li^+^ conduction, but also offer a crucial opportunity to understand the role of electronic density in quasi-solid-state Li metal batteries.
- Donor–acceptor-linked COF electrolyte results in Li^+^ transference number 0.83, high ionic conductivity 6.7 × 10^–4^ S cm^−1^ and excellent cyclic ability in Li metal batteries.
- In situ characterizations, density functional theory calculation and time-of-flight secondary ion mass spectrometry are adopted to expound the mechanism of the rapid migration of Li^+^ in the “donor–acceptor” electrolyte system.

**Supplementary Information:**

The online version contains supplementary material available at 10.1007/s40820-024-01509-y.

## Graphical abstract

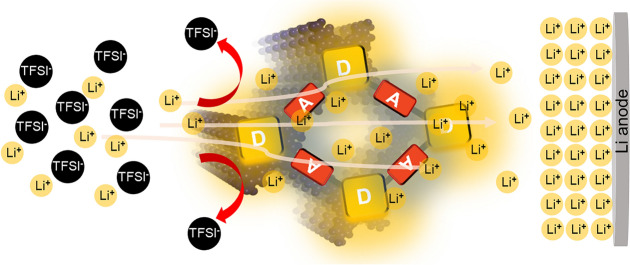

## Introduction

Solid-state Li metal batteries (SSLMBs) equipped solid-state electrolyte (SSE) show high energy density and high safety, as a promising candidate for electric vehicles and energy storage systems [1–3]. However, the SSE is faced with challenges of lowly-ionic conductivity and lowly-selective Li^+^ transportation, which generates Li dendrites risk, unwanted interfacial reaction and bad performance in SSLMBs [4]. These deficiencies are mainly ascribed to the essence that Li^+^ is an electron-deficient species that easily coordinate with anion (TFSI^–^), gave rise to low dissociation degree (low ionic conductivity) and low selectivity (low transference number) [5]. Rich-electron SSE can weaken the strong coordination of Li^+^ and TFSI^–^, enhance Li^+^ migratory kinetics and achieve highly-selective Li^+^ transportation and high conductivity [6]. Nevertheless, designing and constructing novel strategy to regulate electronic concentration of SSE and thoroughly study the connection between electron and Li^+^ have great obstacles.

Donor–acceptor (D–A) system can precisely realize electronic regulation well based on intramolecular charge transfer interactions. Thus, the crucial challenges of Li dendrite growth, bad cyclic stability and unsatisfied Coulombic efficiency (CE) of SSLMBs are avoided by D–A-linked SSEs. However, among the different types of SSEs (sulfide, chloride and oxide electrolytes), the D–A tactic is suitable for polymer electrolyte [7–10]. The reason is that the constituted fragments of polymer skeleton are selected from different donors or acceptors, which the electron transfers from donor to acceptor in the D–A system [11–13]. In addition, the electronic intensity is controlled by linked diverse category donor and acceptor with little effort, bringing about the variation of Li^+^ electronic environment, weakening the electrostatic interaction of Li^+^ and TFSI^–^ and enhancing ionic conductivity, whereas integrating the donor and receptor into polymer that possesses highly periodic structure and well-ordered 1D directional channel for high-efficiency Li^+^ migration and charge transfer is still a big challenge (Scheme S1).

By comparison with other polymers, covalent organic frameworks (COFs) are covalently connected by various organic units that are chose donor/acceptor as linker and have periodic structure, large surface area and 1D directional ion channel, simultaneously [14–17]. In consequence, the D–A-linked COF-based SSE can easily change Li^+^ electronic state, fulfill highly-selective Li^+^ motion and conduction. Unlike published COF-based SSE [18–21], the promising concept with D–A-linked COF-based SSE not only achieves electronic modulation to promote highly-selective Li^+^ migration, enhance ion conductivity and inhibit Li dendrite, but also essentially resolves the crucial issues of slow Li^+^ migration in solid-state Li metal batteries. However, the research of D–A-linked COF-based SSE is quite rare and the relevant mechanism is unclear to date. Therefore, it is enormously significant academic and industrial value to accurate construction D–A-linked COF-based SSE and successful application in solid-state Li metal batteries.

**Fig. 1:**
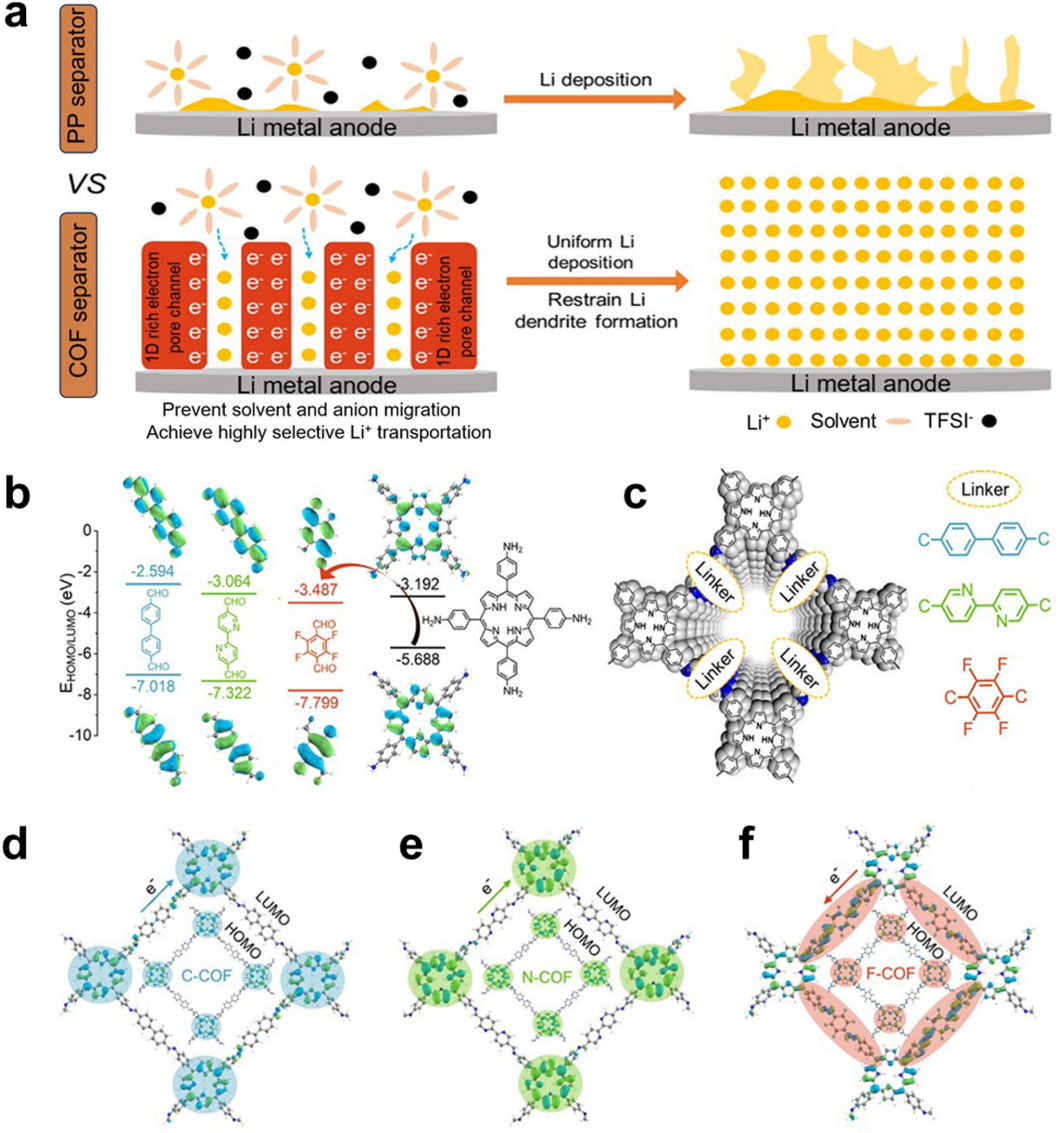
**a** COF SSE with and without 1D rich-electron channel for restraining dendrites. **b** Schematic representation of the LUMO/HOMO energy-level alignments of acceptor units: Bph, Bpy, Tfa and donor TAPP. **c** Different acceptor units-linked covalent organic frameworks. Kohn–Sham LUMOs and HOMOs of **d** C-COF, **e** N-COF and **f** F-COF

In this work, in order to design and elaborate the novel concept, we construct D–A-linked COF-based SSE, diversely electronegative element (C-, N- and F-based) ligand as electron acceptor, the electron-rich tetra(p-amino-phenyl)porphyrin (TAPP)-based as donor. The TAPP ligand shows a number of features with planar and polar molecule, π-electron conjugated system, pyrrolic structure and electronic properties [22–24]. Consequently, establishing D–A system linked by TAPP and highly electronegative element block will enhance the electron transfer efficiency of COF-based SSE and be an important strategy for restraining Li dendrites growth (Fig. 1a). Strongly electronegative element can boost electron transport from donor to acceptor and result in a progressive transference efficiency of Li^+^ (Fig. 1b). Immediately, we confirm elements with different electronegativities on the building units as electron acceptor of target COFs. As demonstrated in Figs. 1b and S1, the calculation results collected from Gaussian reveal that charge transfer from the planar tetragonal TAPP (electron donor) to F-based modified linker (denoted as Tfa, electron acceptor) is easier than other linkers (C-based modified linker: Bph, N-based modified linker: Bpy) [25, 26]. The three linkers represent various electronegativity intensities, which regulates electron density very well. To implement our thought, the COFs structure (denoted as C-COF, N-COF and F-COF) formed by connecting TAPP building units with C, N and F atoms modified linkers (Figs. 1c and S1). It is noteworthy that both the highest occupied molecular orbital (HOMO) and lowest unoccupied molecular orbital (LUMO) of C-COF (Fig. 1d) and N-COF (Fig. 1e) are situated on the TAPP moiety. But for F-COF, the HOMO is located on the TAPP moiety, while the LUMO is transferred to the linkers (Fig. 1f). Such a pronounced D–A property powerfully proves the concept in Fig. 1d–f, which also create a superior chemical environment for Li^+^ adsorption and conduction, with facilitating application in the solid-state Li metal batteries. Therefore, the F-COF-based SSE-assembled quasi-solid-state Li metal batteries show superiorly electrochemical behaviors than substituted C-COF and N-COF.

## Experimental Section

### Synthesis of Various D–A-Linked COFs

A Pyrex tube was charged by TAPP (33.7 mg, 0.05 mmol) and various linkers (Bph: 21.0 mg, 0.1 mmol; Bpy: 21.2 mg, 0.1 mmol; Tfa: 20.6 mg, 0.1 mmol), respectively. Then, a mixed 4.0 mL solution of n-butanol/1,2-dichlorobenzene (1:1, v/v) was added into the tube. Subsequently, the tube was sonicated for 10.0 min, and acetic acid (0.4 mL, 6.0 M) was added to the mixture. The tube was frozen at liquid N_2_ bath and sealed under vacuum. After sealing, the tube was placed in an oven at 120 °C for 3 d. After cooling at room temperature, the yielded solid was collected by filtering and washed with acetone and tetrahydrofuran for several times, respectively. The obtained solid was immersed into anhydrous acetone for 1 d, which the solvent was replaced 4 times per day using fresh solvents. Finally, the active sample was dried at 100 °C for 12 h under vacuum to yield a purple powder C-COF, N-COF and F-COF, respectively.

### Preparation of COF-Based Quasi-Solid-State Electrolyte Membrane

COF powder (200 mg) was immersed in 10 mL LiTFSI propylene carbonate (PC) solution (1 M) for 12 h. Afterward, the powder was obtained by centrifugation, washed by PC to remove the LiTFSI on the surface and dried under vacuum at 120 °C for 12 h. The COF-based SSE membrane was fabricated by thoroughly mixing COF material with 1 wt% polytetrafluoroethylene (PTFE) solution. The obtained powder mixtures were ground for 20 min to form a dough and further rolled into a membrane. The prepared membrane was cut into a small disk (Φ = 16 mm), was immersed in 1 M LiTFSI PC solution for 12 h for activation and was dried at 100 °C for 12 h to remove the moisture residue. The prepared SSE membrane was stored in the glove box for further usage.

## Results and Discussion

### Characterizations of COFs

**Fig. 2:**
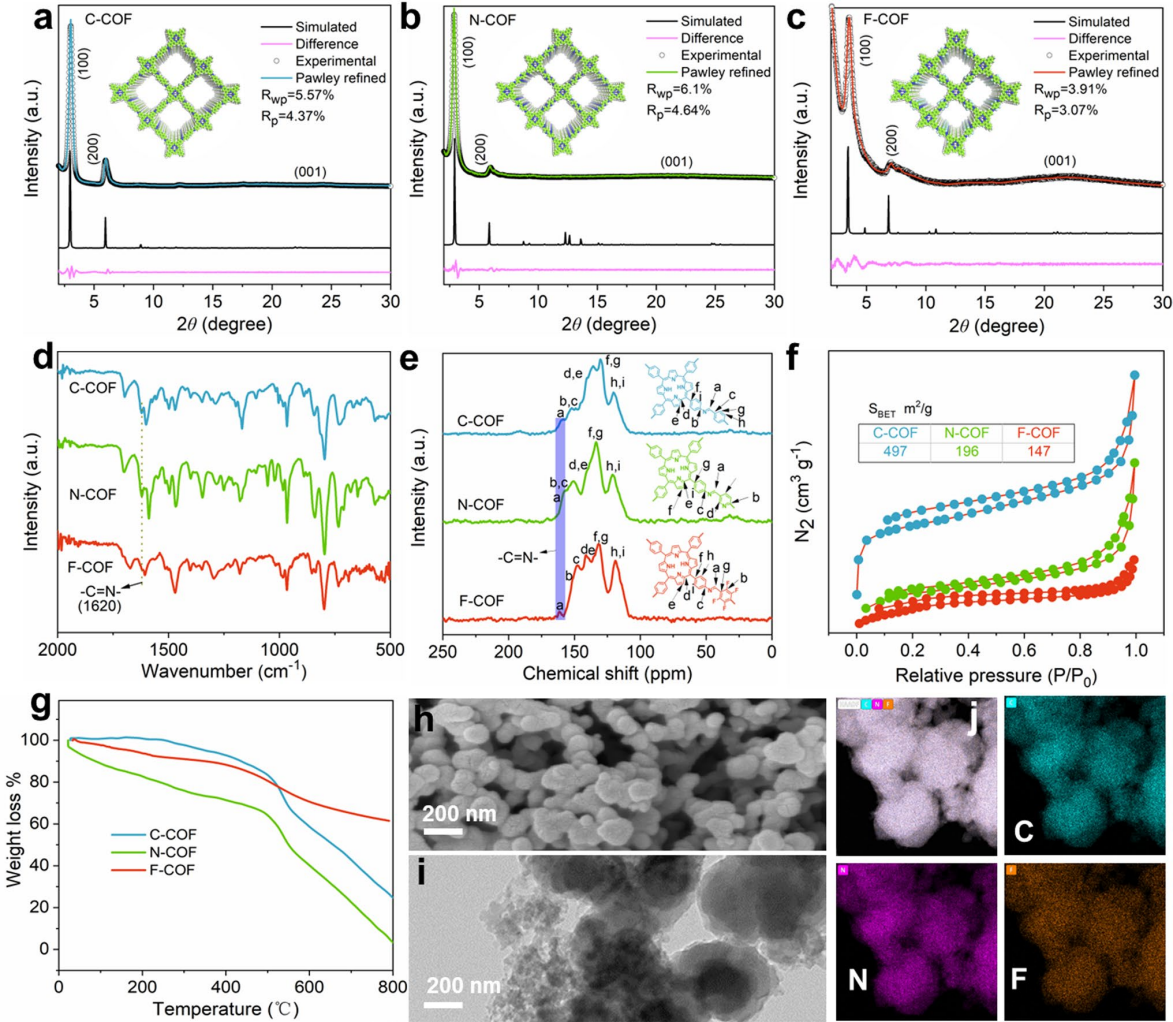
Pawley refinement: **a** C-COF, **b** N-COF and **c** F-COF. **d** FT-IR spectra of C-COF, N-COF and F-COF. **e** Solid-state ^13^C NMR of C-COF, N-COF and F-COF. **f** N_2_ adsorption–desorption curves of C-COF, N-COF and F-COF. **g** TGA curve of C-COF, N-COF and F-COF. **h** SEM, **i** TEM and EDS mappings for F-COF

We design and prepare the porphyrin modified linked COFs with various electron acceptors. The synthetic route of C-COF, N-COF and F-COF is shown in Fig. S1. The crystallinities of prepared COFs materials are studied with powder X-ray diffraction pattern (PXRD). The PXRD patterns show primary characteristic peaks at 2*θ* of 3.04° for C-COF (Fig. 2a), 2.85° for N-COF (Fig. 2b) and 3.54° for F-COF (Fig. 2c), which are ascribed to the (100) face, respectively. Other peaks are assigned to the (200) and (001) faces, which are high accordance with reported works [27, 28]. For further clarification, the effects of imperceptible difference and extremely low values of *R*_wp_ = 3.57%, *R*_p_ = 4.37% for C-COF (Fig. 2a), *R*_wp_ = 6.1%, *R*_p_ = 4.64% for N-COF (Fig. 2b) and *R*_wp_ = 3.91%,* R*_p_ = 3.07% for F-COF (Fig. 2c) can be obtained by the Pawley refinement, which gets a PXRD that is high consistent with experimental data. Moreover, the alternative 2D models with AA and AB stacking are constructed to further elaborate the structure of COFs. Both the experimental PXRD patterns for C-COF (Fig. S2), N-COF (Fig. S3) and F-COF (Fig. S4) are in keeping with the AA stacking model, but not match the possible AB model, meaning the AA stacking drives the structure. In addition, no peaks of starting materials of TAPP, Bph, Bpy and Tfa are found in the PXRD patterns (Figs. S5–S7), demonstrating completely chemical conversion into target COFs. According to the aforementioned analyses, the D–A-linked COFs have prominent crystallinity, which is linked by imine bond via the condensation between NH_2_ group in TAPP and CHO groups in Bph, Bpy and Tfa ligands. As shown in Fig. 2d, the characteristic peaks of C=N are detected by the Fourier-transform infrared (FT-IR) spectrum at wavenumber of 1620 cm^−1^. Moreover, the peaks of NH_2_ and CHO in the starting materials almost are absent in the prepared COFs spectra (Figs. S8–S10), suggesting thorough chemical conversion into COFs [29]. The solid-state ^13^C NMR can further suggest and prove the structure uniformity of C-COF, N-COF and F-COF (Fig. 2e). The C=N and other bonds can be observed in the spectra at chemical shift of 158 ppm, respectively. These peaks are high accordance with FT-IR spectra, implying successful preparation the COFs materials. The porosities of C-COF, N-COF and F-COF are evaluated by the analysis of their N_2_ adsorption isotherms. The C-COF, N-COF and F-COF show Brunauer–Emmett–Teller (BET) surface area of 497, 196 and 147 m^2^ g^−1^, obtained from N_2_ sorption/desorption isotherms at 77 K (Fig. 2f), respectively. The average pore sizes of C-COF, N-COF and F-COF are 2.2, 2.0 and 1.8 nm according to N_2_ sorption/desorption isotherms (Fig. S11). In addition, these COFs have excellent thermostability (Fig. 2g). The morphology of C-COF, N-COF and F-COF is assessed by the scanning electron microscopy (SEM) and transmission electronic microscopy (TEM). The nanorod for C-COF (Fig. S12), nanocube for N-COF (Fig. S13) and nanosphere for F-COF (Fig. S2h) are observed, which further are proved by TEM (Fig. 2i). Moreover, EDS mapping indicates C, N and F elements uniform distribution (Fig. 2j). The favorable crystallinities, porosities and thermostabilities are beneficial for Li^+^ migration [30, 31].

### Electrochemical Performance and Ion Transport Mechanism

**Fig. 3:**
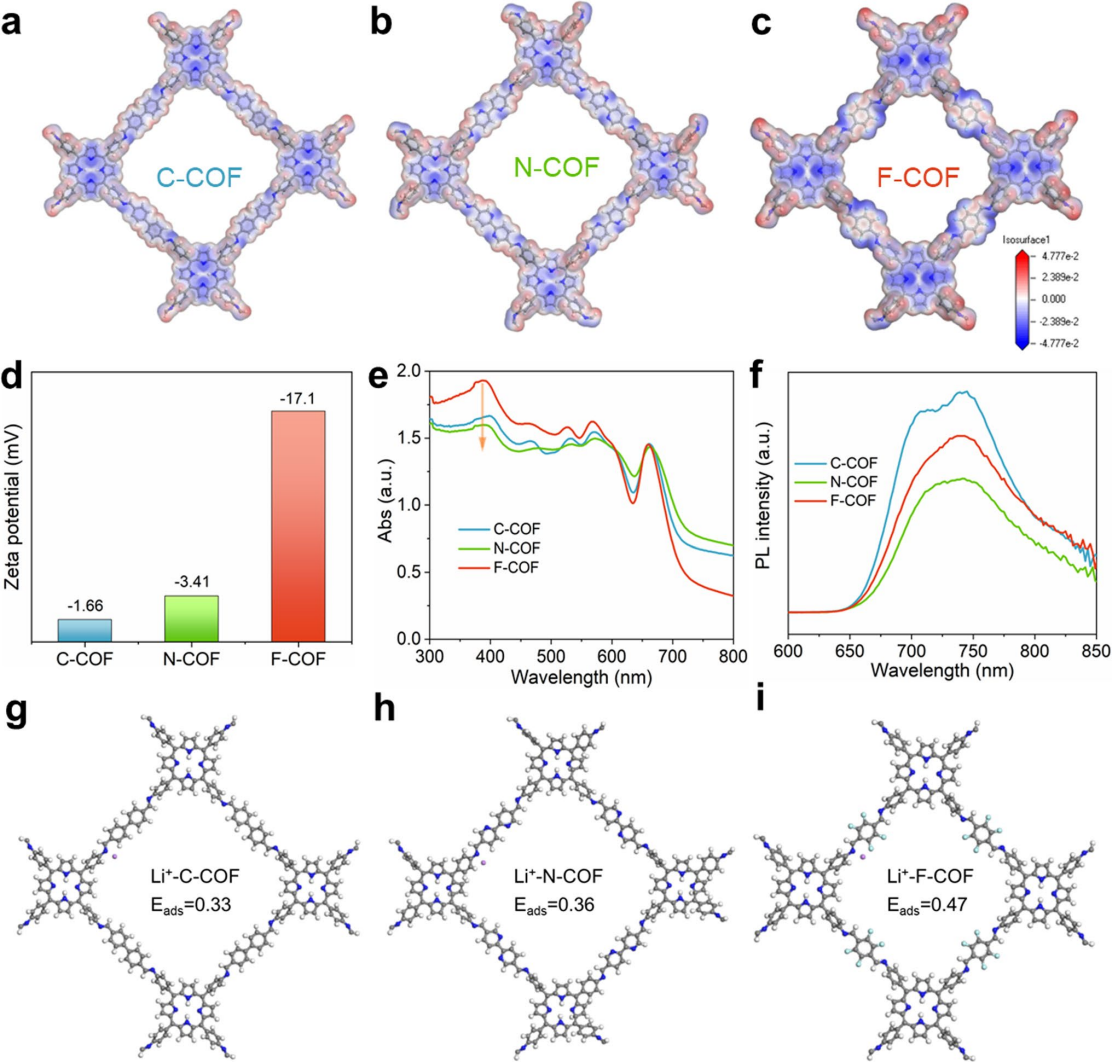
Electrostatic potential mapping: **a** C-COF, **b** N-COF and **c** F-COF. **d** Zeta potential value for C-COF, N-COF and F-COF. **e** UV/Vis absorption curves and **f** PL of C-COF, N-COF and F-COF. Intermolecular interactions between Li^+^ and **g** C-COF, **h** N-COF and **i** F-COF

The microstructure characteristics of C-COF, N-COF and F-COF are extensively studied by theoretical calculation and other tests in order to prove the electronic intensity difference. The electrostatic potential energy of C-COF (Fig. 3a), N-COF (Fig. 3b) and F-COF (Fig. 3c) exhibit optimal electrostatic potential environment, which F-COF has strong electronegativities than C-COF and N-COF. It is caused by the electron migration from TAPP donor to F-based ligand acceptor [25, 26], illustrating F-COF owns superior affinity for positively charged Li^+^. Zeta potential is carried out to further verify the charge amount. The Zeta potential value of Zeta potential for C-COF (− 1.66 mV), N-COF (− 3.43 mV) and F-COF (− 17.1 mV) is obtained in Fig. 3d. The result of Zeta potential is high accordance with electrostatic potential energy, suggesting that F-COF shows forceful adsorption ability toward Li^+^ and rejection for TFSI^−^ because of the existence of electrostatic attraction between Li^+^ and F-COF and electrostatic repulsion between TFSI^−^ and F-COF. Moreover, the difference of electrostatic potential energy and various Zeta potentials demonstrates that the electronic intensity is controlled by linked diverse donor and acceptor in C-COF, N-COF and F-COF. The electronic structures of C-COF, N-COF and F-COF are evaluated using ultraviolet/visible diffuse reflectance spectroscopy (UV/Vis DRS) and photoluminescence (PL) emission spectra. The F-COF has high intensity and redshift absorption compared to C-COF and N-COF. At the same time, the shoulder bands are observed at ~ 720 nm for C-COF, N-COF and F-COF (Fig. 3e). This result exhibits the intramolecular charge transfer between donor and acceptor in COF [32, 33]. The PL emission spectra of C-COF, N-COF and F-COF are shown in Fig. 3f. The moderate PL intensity of F-COF suggests effective charge separation efficiency than C-COF and N-COF. In other words, the F-COF-based SSE has fast Li^+^ transportation kinetics and high conductivity [34]. The detailed affinity between COF-based SSE and Li^+^ is further investigated by adsorption energy calculation (Fig. 3g-i). The binding energy of Li^+^ with C-COF, N-COF and F-COF is assessed to be Li/F-COF (0.47 eV) > Li/N-COF (0.36 eV) > Li/C-COF (0.33 eV). The increased adsorption energy for F-COF is ascribed to the electronegativity-rich F atom, which could preferentially interact with Li^+^ [19]. The attractive ability between F-COF and Li^+^ breaks the strong interaction in TFSI^–^ and Li^+^ and contributes to free Li^+^, thereby achieving highly-selective Li^+^ migration and increasing the Li^+^ transference number.

**Fig. 4:**
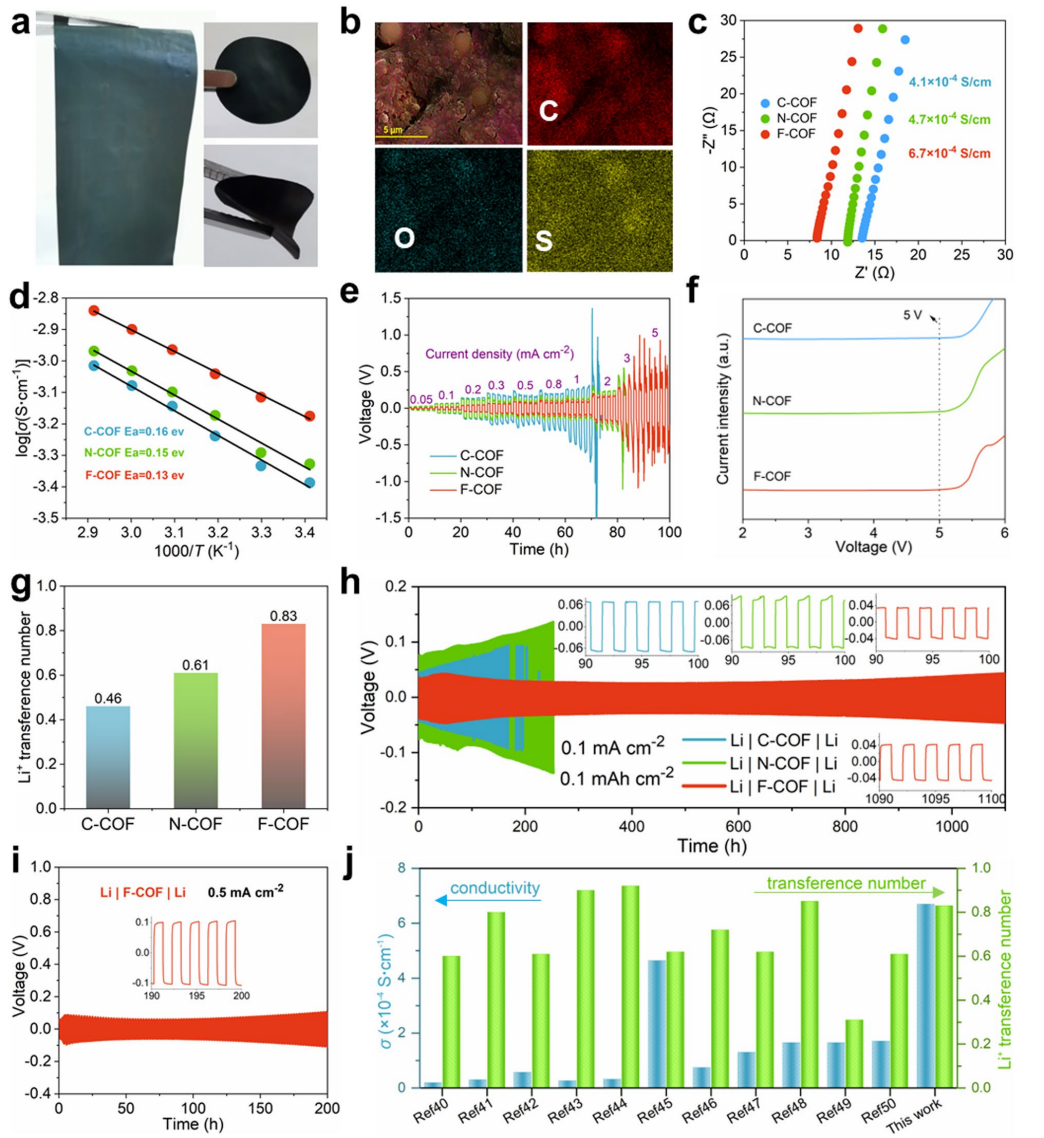
**a** Digital photograph and **b** SEM mapping of F-COF SSE membrane. **c** EIS of C-COF, N-COF and F-COF SSE. **d** *E*a of C-COF, N-COF and F-COF SSE. **e** CCD test and **f** LSV of C-COF, N-COF and F-COF SSE. **g** Li^+^ transference number for C-COF, N-COF and F-COF. **h** Galvanostatic performance of symmetric Li cells with C-COF, N-COF and F-COF SSE at 0.1 mA cm^–2^, insets: enlarged voltage profiles for different times. **i** Galvanostatic cycling of Li|F-COF SSE|Li at current density of 0.5 mA cm^–2^. **j** Conductivity and transference number comparison between F-COF SSE with other reported COF-based SSEs

The flexibility of F-COF SSE is studied in order to explore potential applications in practical processes. A scalable F-COF SSE membrane is fabricated by mixing polytetrafluoroethylene dispersion and is cut into disk with diameter of 16 mm and excellent bending for assembled batteries (Fig. 4a). The thickness of the F-COF electrolytes is measured to be 95 μm by SEM (Fig. S14). More importantly, the elements (representative O and S, originated from LiTFSI) of F-COF SSE membrane are uniformly dispersed (Fig. 4b), indicating no aggregated ion cluster and charge defect in the F-COF SSE. This is beneficial for the Li^+^ conduction [1, 35]. The room-temperature Li^+^ conductivity of C-COF, N-COF and F-COF SSE membrane is evaluated and measured electrochemical impedance spectroscopy (EIS). Figure 4c shows the EIS curves with different impedance values for C-COF (13.5 Ω), N-COF (11.8 Ω) and F-COF (8.3 Ω). The conductivity is calculated to be 4.1 × 10^–4^, 4.7 × 10^–4^ and 6.7 × 10^–4^ S cm^−1^, respectively. The high conductivity is ascribed to outstanding D–A interaction between donor porphyrin and acceptor F atoms, which effectively expedites electron transferring from porphyrin to F-based ligand and enhances Li^+^ kinetics. The activation energy (*Ea*) is assessed by measuring impedances under various temperatures from 20 to 70 °C. The impedances decrease with the temperature increasing for C-COF (Fig. S15), N-COF (Fig. S16) and F-COF SSE (Fig. S17). Therefore, the Arrhenius plots display proportional changes of increased logarithmic ionic conductivity with increasing temperature (Fig. 4d). The *Ea* is determined from the slope of the Arrhenius plot, yielding *Ea* = 0.16 eV for C-COF, 0.15 eV for N-COF and 0.13 eV for F-COF. The remarkable *Ea* proves the existence of directional Li^+^ conduction pathways in these COF SSEs.

The behavior of Li deposition is tested by assembling Li|COF SSE|Li symmetric coin cells at room temperature. Figure 4e shows the relationship of overpotentials with current intensity, representing the value of critical current density (CCD). The CCD of C-COF and N-COF SSEs is 2 and 3 mA cm^–2^, whereas F-COF is 5 mA cm^–2^. Higher CCD suggests a good performance of inhibiting Li dendrite and operation at high rate in solid-state batteries [36]. The high CCD in F-COF is ascribed to the stable framework structure that strong electronegativity F atom can enhance the stability. Electrochemical stability window of C-COF, N-COF and F-COF SSE membrane is characterized by testing linear sweep voltammetry. Both the C-COF, N-COF and F-COF SSEs have high stability window (> 5 V), which indicates the prepared COF-based SSE can match high-voltage cathode. The highly electrochemical stability window is attributed to rich-electron channels that enhance the antioxidant ability. The Li^+^ transference number (Fig. 4g) can be obtained from the AC impedance spectrum and current–time curve of C-COF (Fig. S18), N-COF (Fig. S19) and F-COF SSE (Fig. S20), yielding 0.46 for C-COF, 0.61 for N-COF and 0.83 for F-COF, respectively. The high Li^+^ transference number for F-COF is attributed to the rich-density electronegative environment that inhibits TFSI^−^ migration and can effectively delay the time of dendrite formation according to Sand’s model [37]. The interfacial compatibility between COF SSE and Li anode is studied by Li|COF SSE|Li symmetric coin cells. Figure 4h presents the curve of overpotentials toward time at current density with 0.1 mA cm^–2^ (0.1 mAh cm^–2^). The overpotentials of Li|C-COF SSE|Li and Li|N-COF SSE|Li cells increase with the tested time, indicating poor interfacial stability and dendrite growth. However, the Li|F-COF SSE|Li cell has low overpotential value with ~ 40 mV and the stable voltage hysteresis can be more than 1000 h. Furthermore, the Li|F-COF SSE|Li symmetric cell can be more than 200 h at high current density with 0.5 mA cm^–2^ and 0.5 mAh cm^–2^ (Fig. 4i). In consequence, the F-COF SSE has high Li conductivity, high Li^+^ transference number, low *Ea*, broad electrochemical stable window and remarkably interfacial stability and compatibility than C-COF and N-COF SSE. These highlighted performances are ascribed to the outstanding D–A interaction between TAPP and F atoms in F-COF skeleton, which effectively expedites electron transferring from TAPP to F-based ligand and reduces Li^+^ migration energy. Furthermore, the conductivity and Li^+^ transference number of F-COF SSE are compared with recently reported COF-based SSE, which shows better behaviors (Fig. 4j) [38–48].

**Fig. 5:**
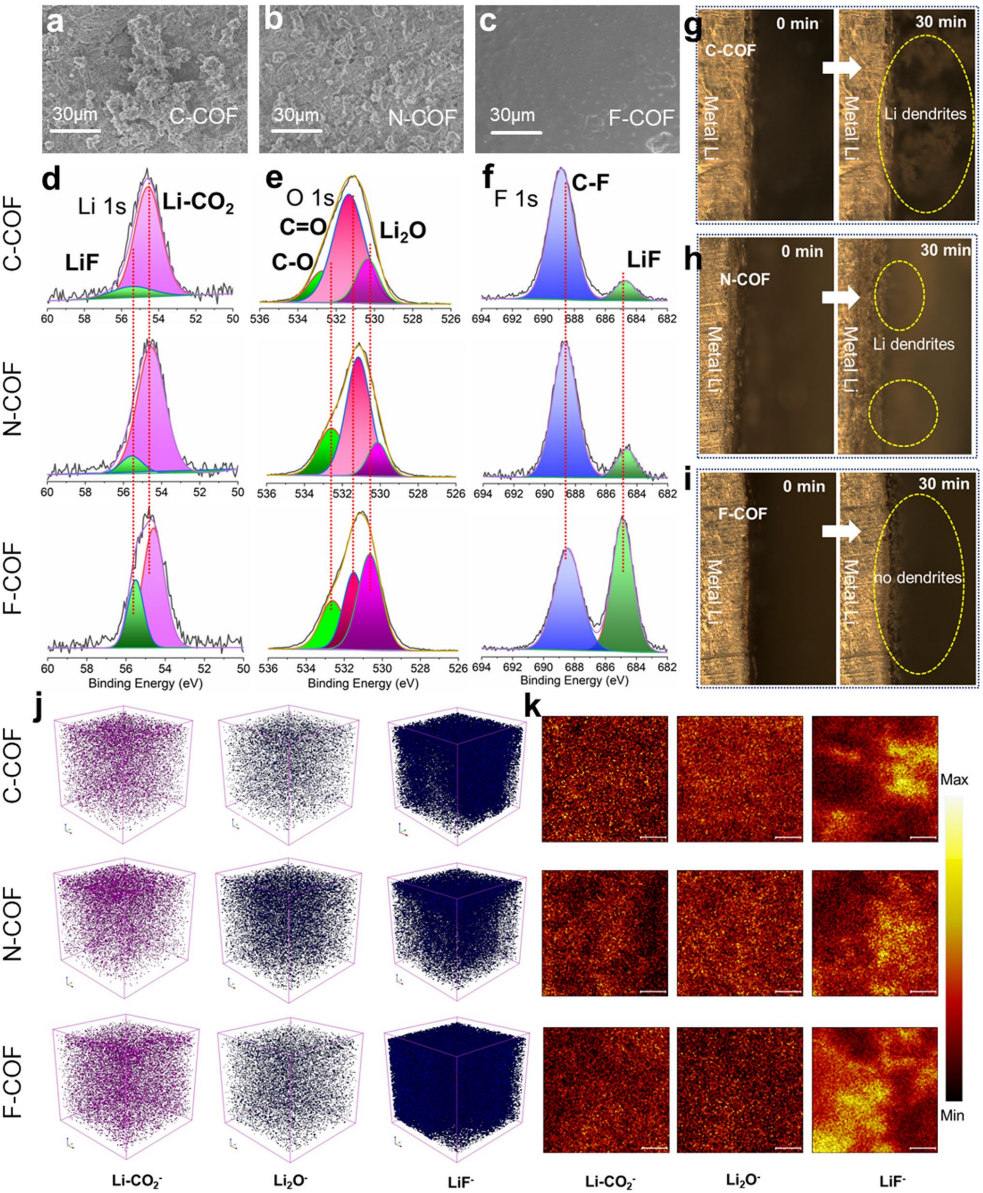
SEM images of Li anode after cycling: **a** C-COF, **b** N-COF and **c** F-COF. XPS spectra analysis of the SEIs: **d** Li|C-COF SSE|Li, **e** Li|N-COF SSE|Li, **f** Li|F-COF SSE|Li. In situ optical microscopy observations of the Li dendrite growth process: **g** Li|C-COF SSE|Li, **h** Li|N-COF SSE|Li, **i** Li|F-COF SSE|Li at current density of 1 mA cm^–2^. **j** ToF–SIMS 3D and **k** 2D spectra of Li-CO_2_^–^, Li_2_O^–^ and LiF^-^ for Li anode surface of Li|C-COF SSE|Li, Li|N-COF SSE|Li, Li|F-COF SSE|Li cells

The interfacial stability between C-COF, N-COF and F-COF with Li anode is thoroughly studied in the Li|COF SSE|Li symmetric cell. The SEM images of metal Li after cycling measurements symmetric cell are studied. As shown in Fig. 5a–c, it is obvious to observe the dendrite or dead Li on the surface of metal Li for C-COF and N-COF. However, the F-COF-based solid-state battery shows smooth surface and the dendrite is almost not found. These results suggest that F-COF has exceptional strength of hindering Li dendrite, promotes Li^+^ uniform deposition and accelerates Li^+^ migrated kinetics. The superficial chemical constituents of Li anode after cycles are monitored using X-ray photoelectron spectroscopy (XPS) in order to assess the interfacial stability and electrolyte stability. As shown in Fig. 5d-f, the high-resolution Li 1*s*, O 1*s* and F 1*s* of C-COF, N-COF and F-COF-based solid-state batteries show SEI ingredients of LiF, Li-CO_2_ and Li_2_O [49]. However, it is obvious to obverse that the intensity of these peaks is stronger in the F-COF-based solid-state battery, suggesting that F-COF SSE has outstanding interfacial regulated ability. Moreover, the F 1*s* XPS spectrum of bare C-COF, N-COF and F-COF displays C-F_3_ and LiF species, which are from the decomposition of TFSI^−^. However, the F-COF SSE shows high content of C-F_3_ and LiF demonstrating good SEI stability. In consequence, the rich-electron F-COF SSE can restrain TFSI^−^ transportation and improve the overall performance of F-COF**-**based solid-state batteries. We further assess the ability of restraining dendrite growth for C-COF, N-COF and F-COF SSE. In situ optical microscopy is carried out to observe the Li deposition and dendrite procedures in the Li metal symmetric cell under current density of 1 mA cm^–2^. As displayed in Fig. 5g–h, for the bare C-COF and N-COF, the Li dendrite can be obviously found when the battery is run. However, for F-COF SSE (Fig. 5i), almost no dendrite is found at any time, and the surface of Li is without changes. Consequently, the F-COF is a promising SSE membrane for achieving highly-selective Li^+^ transportation and dendrite-free Li metal solid-state batteries. Finally, the signals and thicknesses of LiF^−^, Li-CO_2_^−^ and Li_2_O^−^ derived SEI from C-COF, N-COF and F-COF SSE are studied by time-of-flight secondary ion mass spectrometry (ToF–SIMS). The F-COF SSE shows uniform, profound and abundant SSE, which the signals and depth are significantly stronger than C-COF and N-COF. This is corresponding to the XPS analysis. The above results indicate that the TFSI^−^ is freely mobile in the C-COF and N-COF SSEs, impeding Li^+^ migration [50, 51].

**Fig. 6:**
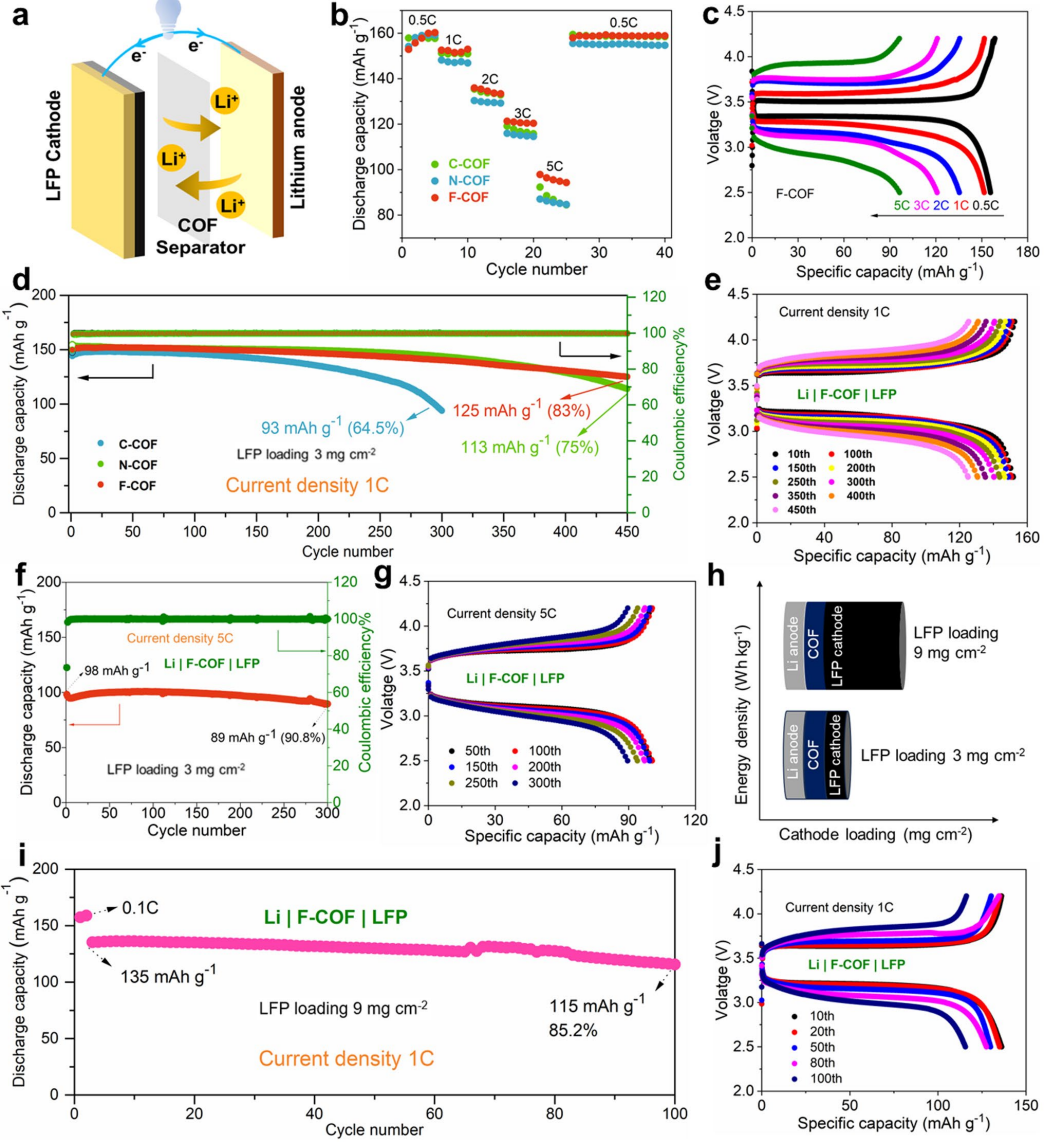
**a** Model of solid-state COF-based LFP|Li metal battery. **b** Rate performance of Li|C-COF SSE|LFP, Li|N-COF SSE|LFP and Li|F-COF SSE|LFP. **c** Galvanostatic charging/discharging curves of Li|F-COF SSE|LFP at various rates; **d** cycling stability of Li|C-COF SSE|LFP, Li|N-COF SSE|LFP and Li|F-COF SSE|LFP at 1C. **e** Charging/discharging curves of Li|F-COF SSE|LFP at various cycles. **f** Cycling stability of Li|F-COF SSE|LFP at 5C. **g** Charging/discharging curves of Li|F-COF SSE|LFP at various cycles. **h** Schematic illustration of energy density in solid-state Li metal batter with different LFP loadings. **i** Cycling stability of Li|F-COF SSE|LFP at 1C with 9 mg cm^–2^ LFP loading. **j** Charging/discharging curves of Li|F-COF SSE|LFP at various cycles with 9 mg cm^–2^ LFP loading

We farther appraise the feasibleness of prepared COF SSE separators via the assembled full battery, LiFePO_4_ (LFP) as cathode, COF SSE as separators and metal Li as anode (Fig. 6a). The Li|F-COF SSE|LFP has better rate performance than Li|C-COF SSE|LFP and Li|N-COF SSE|LFP batteries (Fig. 6b). The discharge capacity with 160.2, 152.3, 133.34, 120.3 and 95.4 mAh g^–1^ at 0.5, 1, 2, 3 and 5 C in Li|F-COF SSE|LFP (Fig. 6c) is higher than Li|C-COF SSE|LFP (Fig. S21) and Li|C-COF SSE|LFP (Fig. S22). In addition, the Li|F-COF SSE|LFP has better cyclic stability at 1C than Li|C-COF SSE|LFP and Li|C-COF SSE|LFP (Fig. 6d). The capacity retention ration for these quasi-solid-state batteries is 64.5% (300 cycles), 75% (450 cycles) and 83% (450 cycles). The discharge capacity after 450 cycles is 125 mAh g^–1^ for Li|F-COF SSE|LFP (Fig. 6e), higher than 93 mAh g^–1^ for Li|C-COF SSE|LFP (Fig. S23) and 113 mAh g^–1^ for Li|N-COF SSE|LFP (Fig. S24). We also assess the Li|F-COF SSE|LFP quasi-solid-state battery fast-charge/discharge performance, which shows good capacity retention ration of 90.8% after 300 cycles at high current density 5C (Fig. 6f). The discharge capacity with 89 mAh g^–1^ can be obtained after 300 cycles at 5C (Fig. 6g), indicating that the Li|F-COF SSE|LFP solid-state battery has great potential in power electric vehicle. The high-loading cathode is the critical factor for high-energy solid-state Li metal batteries (Fig. 6h) [10]. The Li|F-COF SSE|LFP solid-state battery with high-loading LFP (9 mg cm^–2^) can obtain satisfactory cyclic stability 85.2% (Fig. 6i) and discharge capacity of 115 mAh g^–1^ (Fig. 6j) at 1C after 100 cycles. The full battery tests can further prove that the D–A effects play a crucial role in quasi-solid-state batteries.

**Fig. 7:**
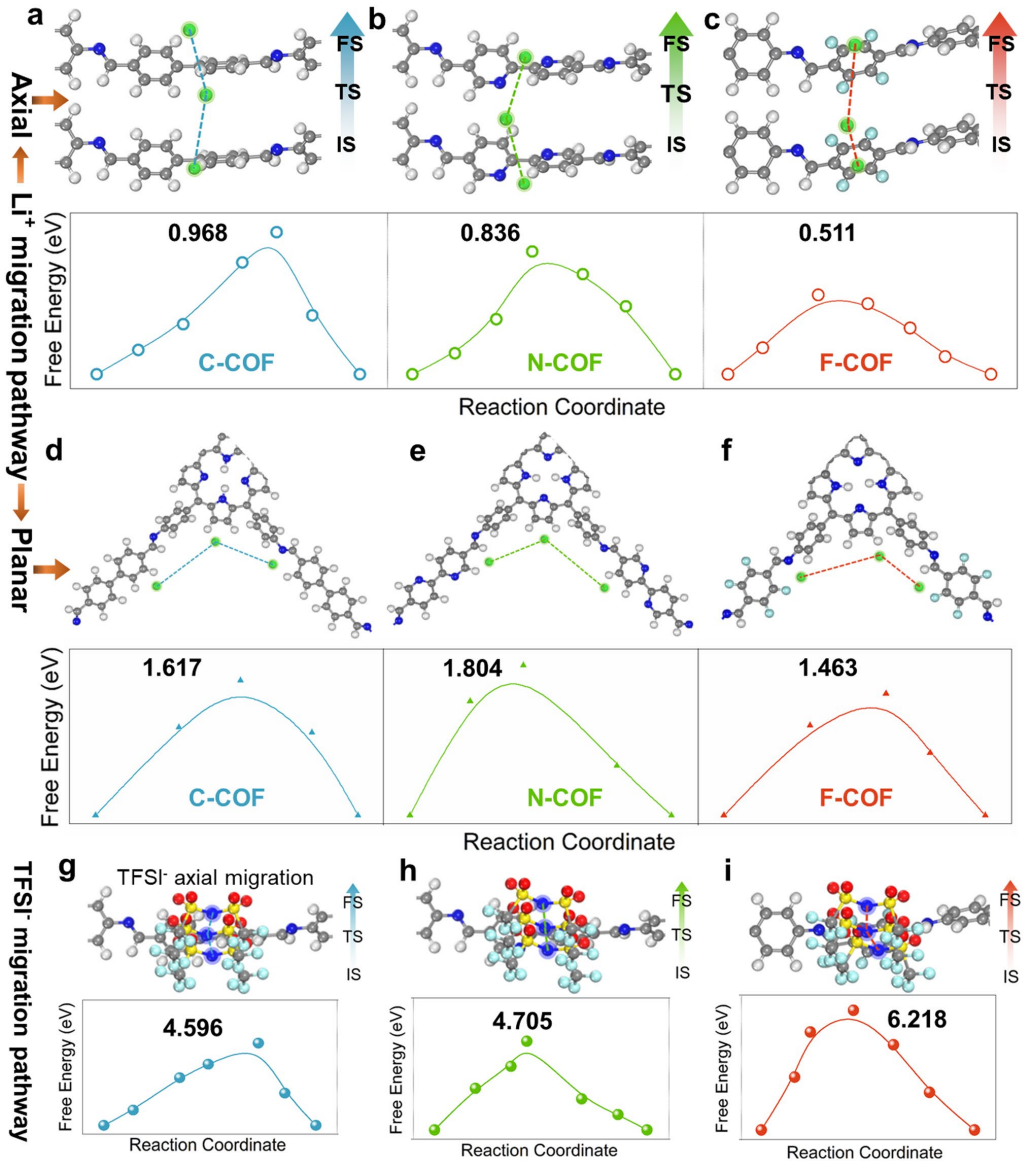
Theoretical calculation for ion migration: Li^+^ migration behaviors inside the pore with corresponding energy diagrams: **a** C-COF, **b** N-COF and **c** F-COF SSE. The initial states, transition states and final states are abbreviated as IS, TS and FS; Li^+^ migration behaviors inside the planar with corresponding energy diagrams: **d** C-COF, **e** N-COF and **f** F-COF SSE. The initial states, transition states and final states are abbreviated as IS, TS and FS; TFSI^–^ migration behaviors inside the pore with corresponding energy diagrams: **g** C-COF, **h** N-COF and **i** F-COF SSE

The driving force of Li^+^ diffusion behaviors in C-COF, N-COF and F-COF SSE is traced through theoretical calculation. More specifically, Li^+^ migration barrier (*E*_m_) along the axial pathway of F-COF requires a lower migration barrier (*E*_m_ = 0.511 eV) compared to that in the C-COF and N-COF (*E*_m_ = 0.968 and 0.836 eV), as shown in Fig. 7a–c, respectively. This optimal axial Li^+^ migration behavior could be caused by the preferred adsorption and desorption properties, which is promoted by the F atoms (light blue-colored) of Tfa units. Note that the Li^+^ diffusion energy in the planar pathway of C-COF, N-COF and F-COF SSE is higher than that in the axial one, which should be attributed to the longer hopping distances (Fig. 7d–f). These results powerfully demonstrate that the adsorbed Li^+^ prefers conduction along the stacked pores of COF rather than accumulating at Li surface [41]. Compared with Li^+^, the TFSI^–^ in the C-COF, N-COF and F-COF SSE needs much bigger axial migration energy to support it diffusion, and reaching an astonishing 4.596 in C-COF, 4.705 in N-COF and 6.218 eV in F-COF SSE (Fig. 7g–i). It also indirectly suggests that Li^+^ is the principal transport species in F-COF aperture and can achieve highly-selective Li^+^ transportation and dendrite-free solid-state Li metal battery.

## Conclusions

A unique strategy for Li conduction is successfully developed based on electronic modulation engineering through donor–acceptor-linked COFs solid-state electrolyte in this work. Due to the F-COF linked by porphyrin units with plentiful π-conjugated systems and F atoms, the prominent D–A interaction is reasonably designed and achieves rich-electron channels. Accordingly, the F-COF solid-state electrolyte creates a favorable environment for the Li^+^ migration, which is beneficial to promote the Li^+^ uniform deposition and has salient performance for Li dendrite-free. We expect that this technology of designing and synthesizing functional solid-state electrolyte has potential applications and values for achieving Li dendrite-free in solid-state Li metal battery accelerate the advancements of COFs in the energy fields as well.

## Supplementary Information

Below is the link to the electronic supplementary material.

Supplementary file1 (DOCX 4261 KB)
