# Supplementary material for: Constructing Donor–Acceptor-Linked COFs Electrolytes to Regulate Electron Density and Accelerate the Li+ Migration in Quasi-Solid-State Battery
Source: Nanomicro Lett. 2024 Sep 26;17:21. doi: 10.1007/s40820-024-01509-y (PMC11427627; doi:10.1007/s40820-024-01509-y)
Supplement: Supplementary file 1 — Supplementary file1 (DOCX 4261 KB) [file 40820_2024_1509_MOESM1_ESM.docx]

Supporting Information for

**Constructing Donor−Acceptor Linked COFs Electrolytes to Regulate Electron Density and Accelerate the Li^+^ Migration in Quasi-Solid-State Battery**

Genfu Zhao^1^, Hang Ma^2^, Conghui Zhang^1^, Yongxin Yang^1^, Shuyuan Yu^1^, Haiye Zhu^1^, Yongjiang Sun^1^, Hong Guo^1,3,^*

^1^ School of Materials and Energy, International Joint Research Center for Advanced Energy Materials of Yunnan Province, Yunnan University, Kunming 650091, P. R. China.

^2^ R & D Center, Yunnan Yuntianhua Co., Ltd., Kunming 650228, P. R. China

^3^ Southwest United Graduate School, Kunming 650091, P. R. China

*Corresponding author. E-mail: [guohong@ynu.edu.cn](mailto:guohong@ynu.edu.cn) (Hong Guo)

**S1 Materials**

All the starting materials and solvents, unless otherwise noted, are used without purification. Tetra(p-amino-phenyl)porphyrin (TAPP), biphenyl dialdehyde (Bph), bipyridine-dialdehyde (Bpy) and 2,3,5,6-tetrafluoroterephthaldehyde (Tfa) were purchased from the Jilin Chinese Academy of Sciences-Yanshen Technology Co., Ltd. 1,2-Dichlorobenzene, n-butanol, tetrahydrofuran, polyvinylidene fluoride (PVDF), N-methyl pyrrolidone (NMP) and CH_3_COOH (6.0 M) were obtained from Shanghai Titan Scientific Co. Ltd. LFP and carbon black (Super P) were obtained from Hefei Kejing Materials Technology Co., Ltd.

**S2 Characterized Instruments**

The crystallinity of obtained COFs was measured by powder X-ray diffraction (PXRD) and recorded using a Bruker D8 Advance X-ray diffractometer at 40 kV and 40 mA over 2° to 30° 2*θ* range with a Cu Kα radiation. The microstructures of prepared materials were studied by scanning electron microscope (SEM) (AMRAY 1000B) and transmission electron micrographs images (TEM, JEM-2010). The porosity of COFs was investigated by nitrogen adsorption–desorption isotherms measured at 77 K on an Autosorb IQ2 absorptiometer (Quantachrome Instruments). The thermostability of COFs were measured by thermogravimetric analysis (TGA) carried out on a Netzch STA449F3 analyzer under N_2_ atmosphere at a heating rate of 10 °C/min from 25 to 800 °C. Solid-state nuclear magnetic resonance (NMR) experiments were conducted using an Agilent VNMRS 600 MHz NMR spectrometer at room temperature. The galvanostatic discharge/charge tests were carried out by using Neware CT-4008-5V50mA-164 laboratory instrument at different current densities.

**S3 Density Functional Theory (DFT) Calculations**

Density functional theory was carried out using the Ab-initio Simulation Package (VASP).^1^ The Generalized Gradient Approximation (GGA) within the Perdew-Burke-Ernzerh (PBE) functional was employed.^2^ The projector augmented wave (PAW)^3^ method and plane wave basis sets were used. And the plane wave cut-off energy was set as 450 eV. Structural optimizations were performed by minimizing the forces on all the atoms to below 0.05 eV·Å^-1^ and the energy to below 10^-5^ eV. Γ point was used in the k-point sampling. The Brillouin zone was sampled using 2 × 1 × 1 for three structures. Furthermore, to determine the diffusion energy barrier and the minimum energy pathways for Li diffusion in COF, the nudged elastic band (NEB)^4^ method was performed.

**S4 Preparation of LFP Solid-state Battery**

The LFP powders, super P and poly(vinyl difluoride) (PVDF) were mixed at a weight ratio of 90:5:5 in NMP solvent to form a homogeneous slurry and then coated on Al foil. The electrodes were dried at 80 °C for 10 h under vacuum. It was then punched into discs (Φ = 14.0 mm). The active cathode materials loading was about 3.0 and 9.0 mg cm^-2^. Solid-state battery (Li|COF SSE|LFP) was assembled by LFP as cathode, COF as separator, metal Li as anode. The galvanostatic charge/discharge tests of solid-state battery (Li|COF SSE|LFP) are recorded in battery testing system (using Neware CT-4008-5V50mA-164 laboratory instrument) with the cut-off voltages of 2.5–4.2 V under various current density at room temperature.

**S5 Li^+^ Transference Number**

Li^+^ transference number (*t*_Li⁺_) was evaluated using a potentiostatic polarization method at room temperature. The DC current flowing through the Li/Li symmetric cell and the AC impedance of the cell before and after polarization were measured to determine the *t*_Li⁺_ value of COFs according to following equation:

*t*_Li⁺_ $=\frac{Iss(\triangle V-I0R0)}{I0(\triangle V-IssRss)}$

where I_0_ is the initial current, I_SS_ represents the steady-state current, ∆V symbolizes the applied potential, R_0_ and R_SS_ are the interfacial resistances before and after the polarization, respectively.

**S6 Li^+^ Conductivities**

Li^+^ conductivities σ of C-COF, N-COF and F-COF were determined based on EIS measurements on an electrochemical workstation with a frequency of 10^5^ Hz to 0.1 Hz and a temperature control system. The ionic conductivities were then calculated according to following equation:

*σ* $=\frac{l}{SR}$

where l (cm) is the thickness of the electrolyte, S (cm^2^) is the area of the electrolyte, and R (Ω) is the bulk resistance.

**S7 Supplementary Figures**


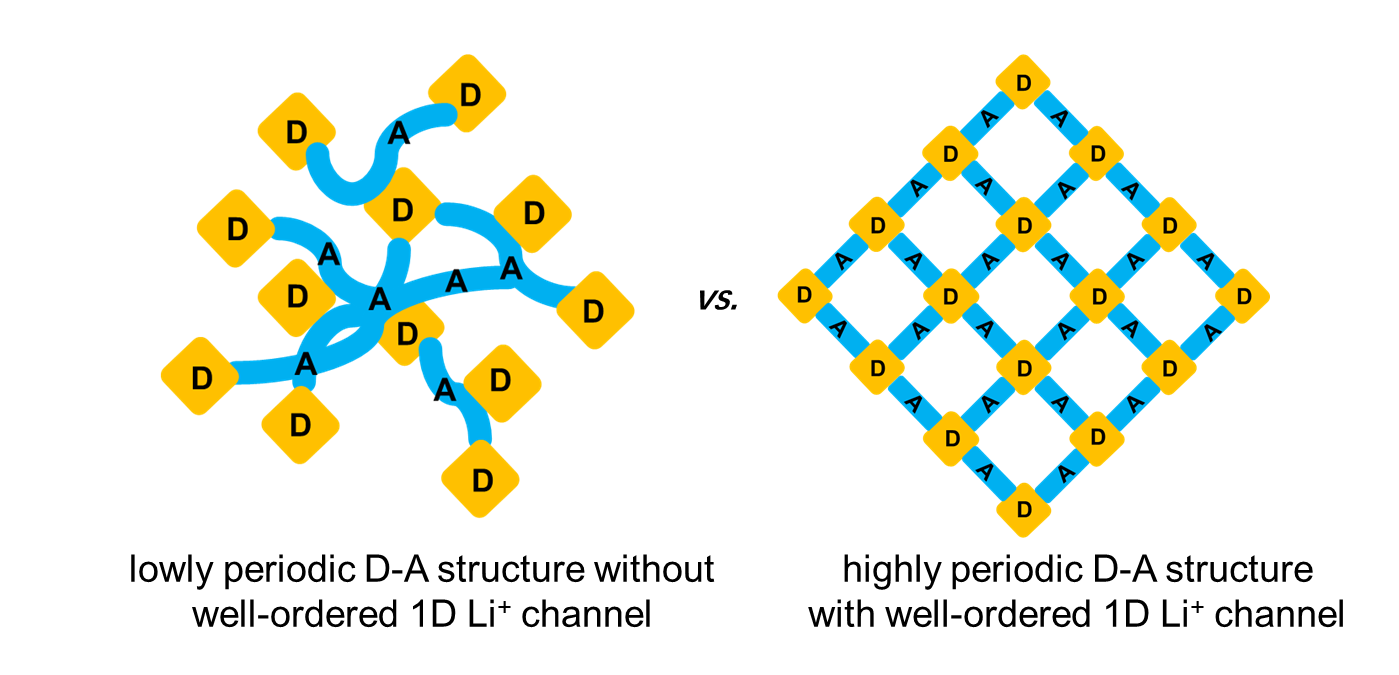


**Scheme S1** Schematic illustration of lowly and highly periodic D-A strucutre


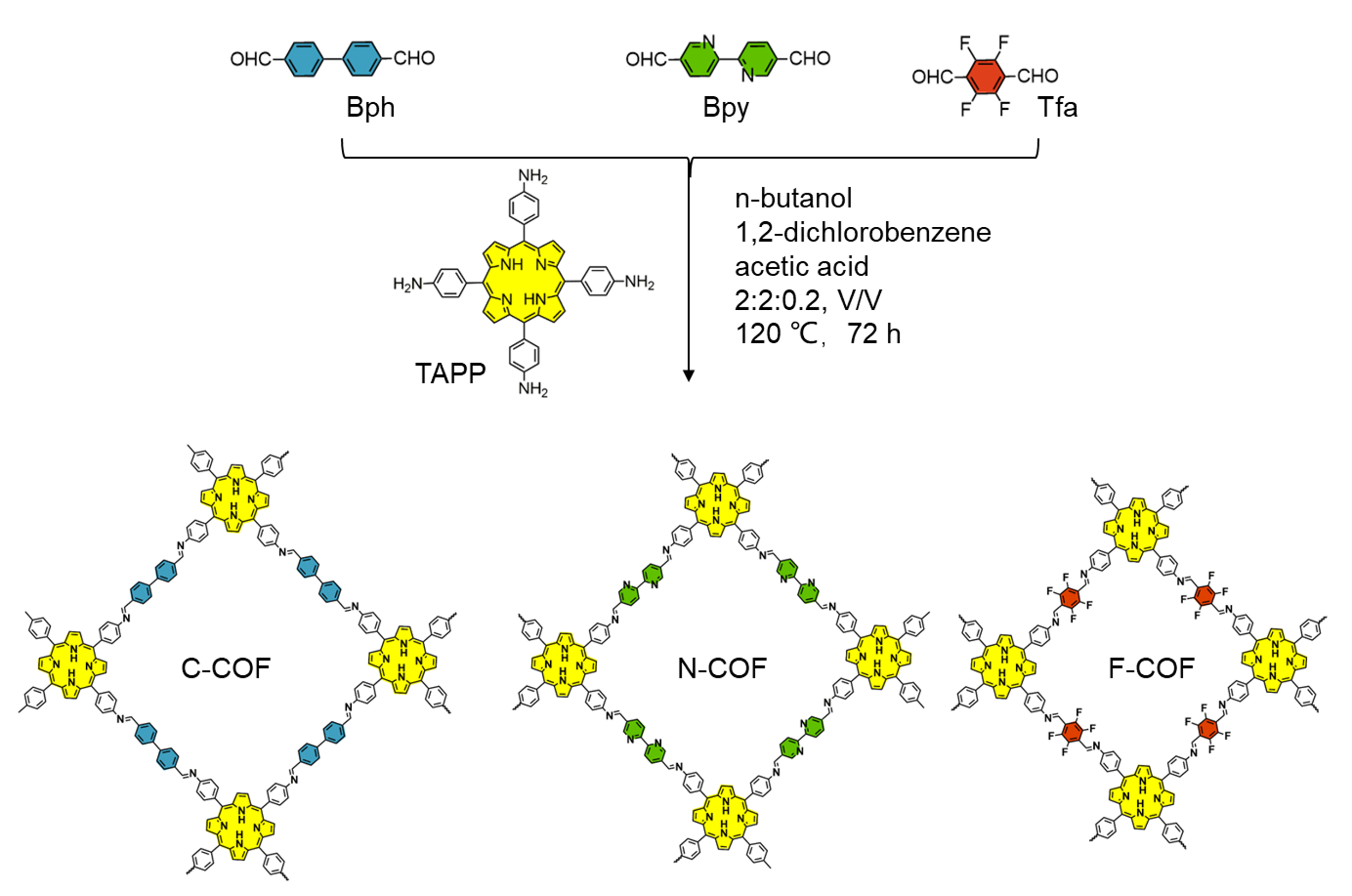


**Fig. S1** The synthetic route of C-COF, N-COF and F-COF, and the electron transfer


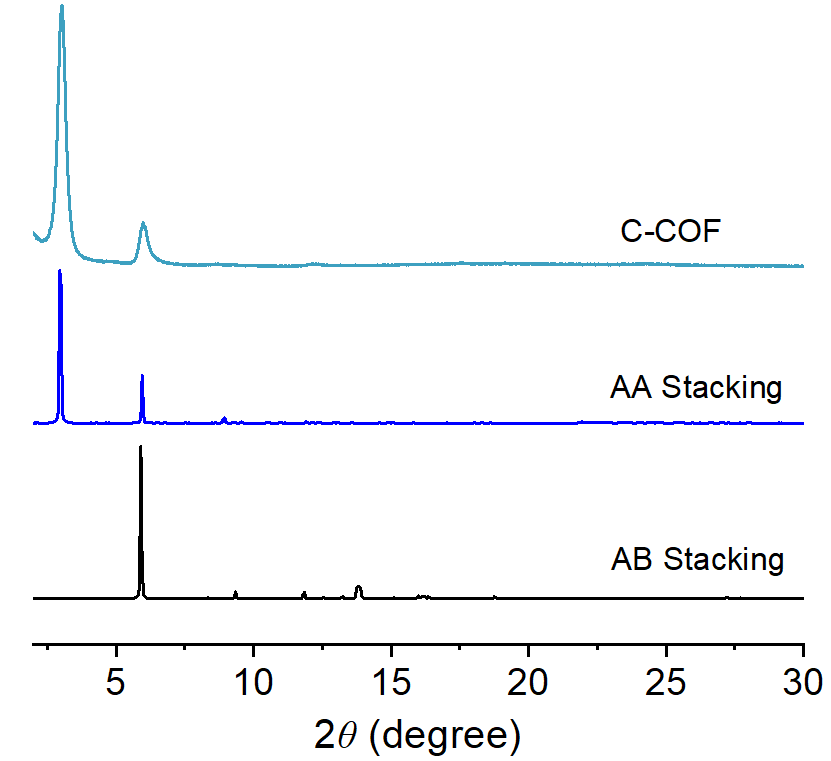


**Fig. S2** Calculated PXRD patterns of AA and AB and experimental results for C-COF


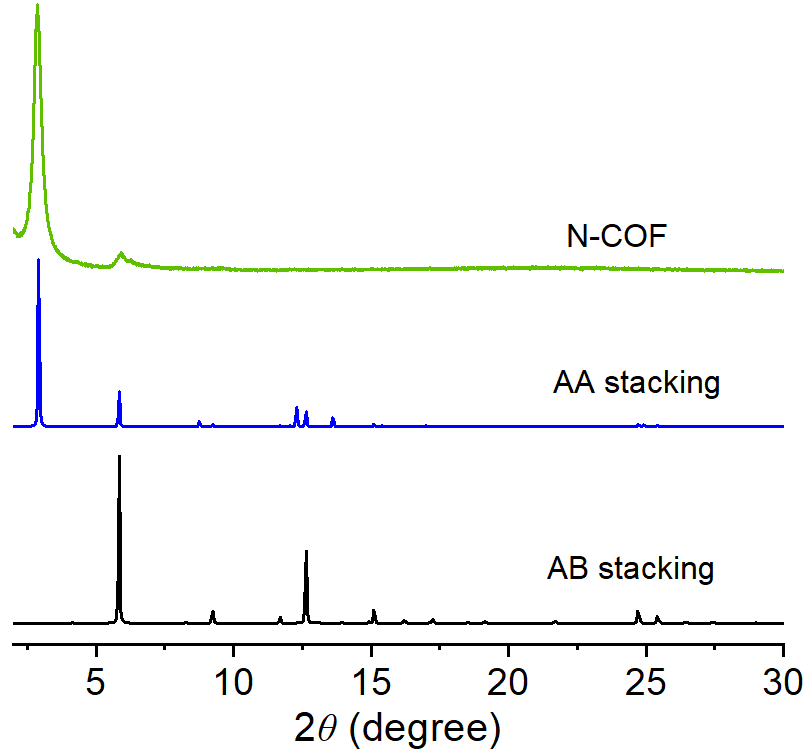


**Fig. S3** Calculated PXRD patterns of AA and AB and experimental results for N-COF


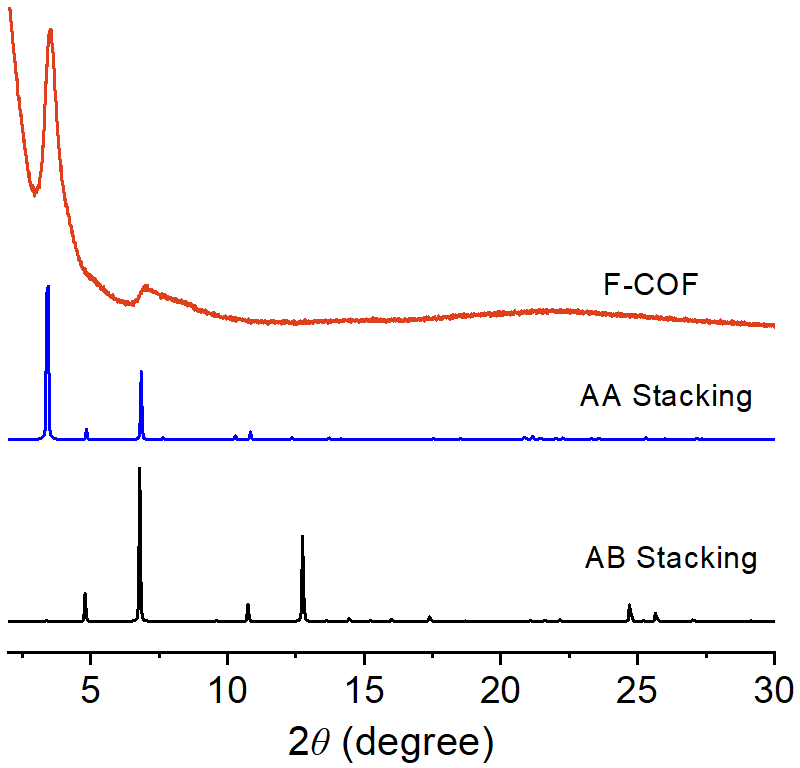


**Fig. S4** Calculated PXRD patterns of AA and AB and experimental results for F-COF


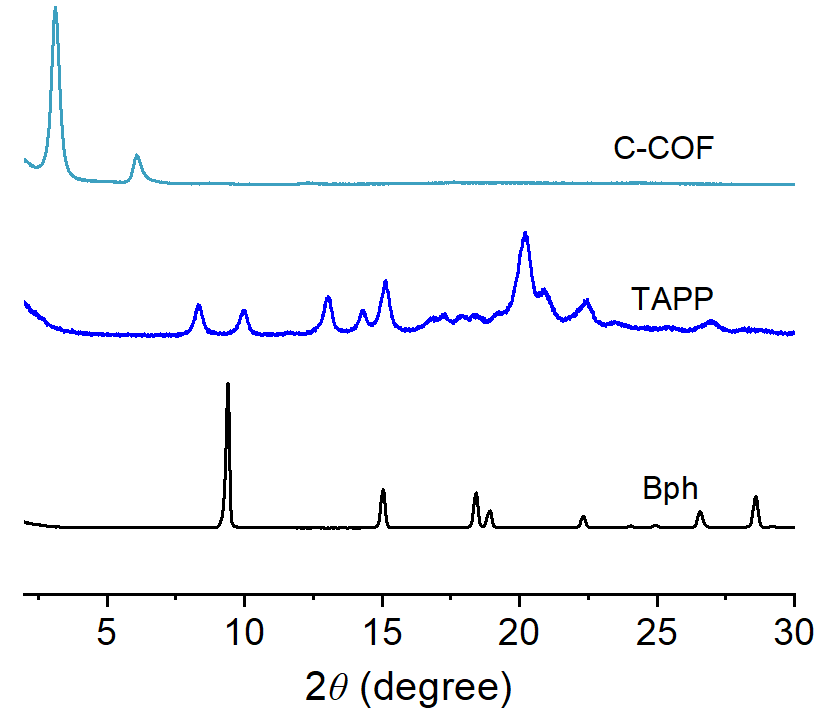


**Fig. S5** PXRD patterns of synthesized C-COF and starting materials of Bph and TAPP


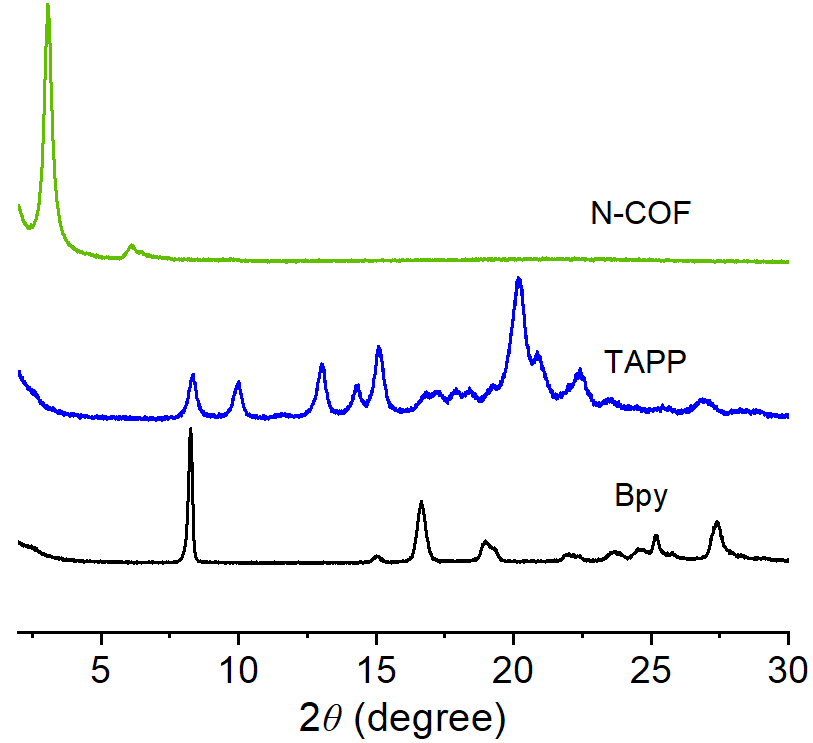


**Fig. S6** PXRD patterns of synthesized N-COF and starting materials of Bpy and TAPP

**
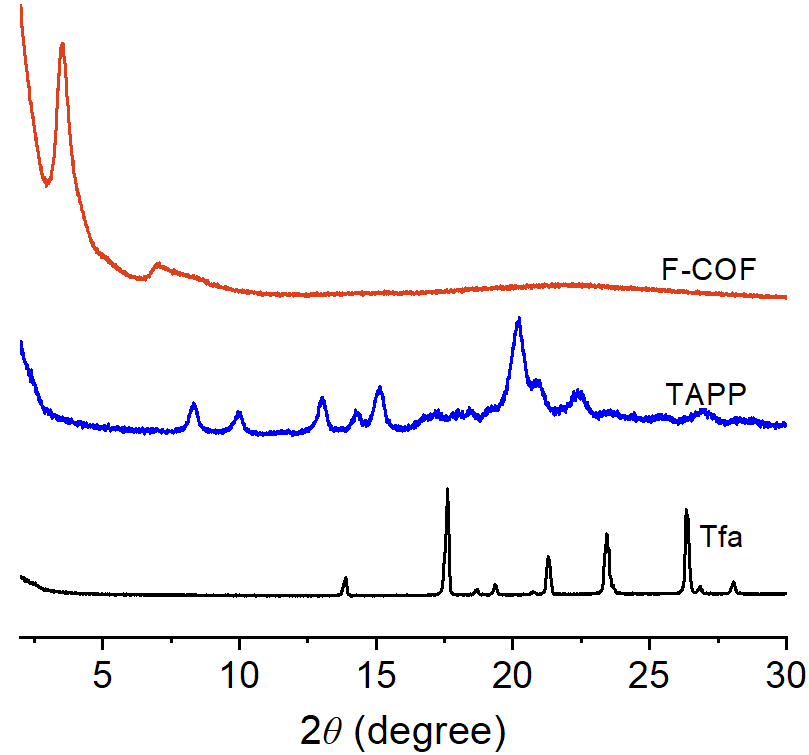
**

**Fig. S7** PXRD patterns of synthesized F-COF and starting materials of Tfa and TAPP

**
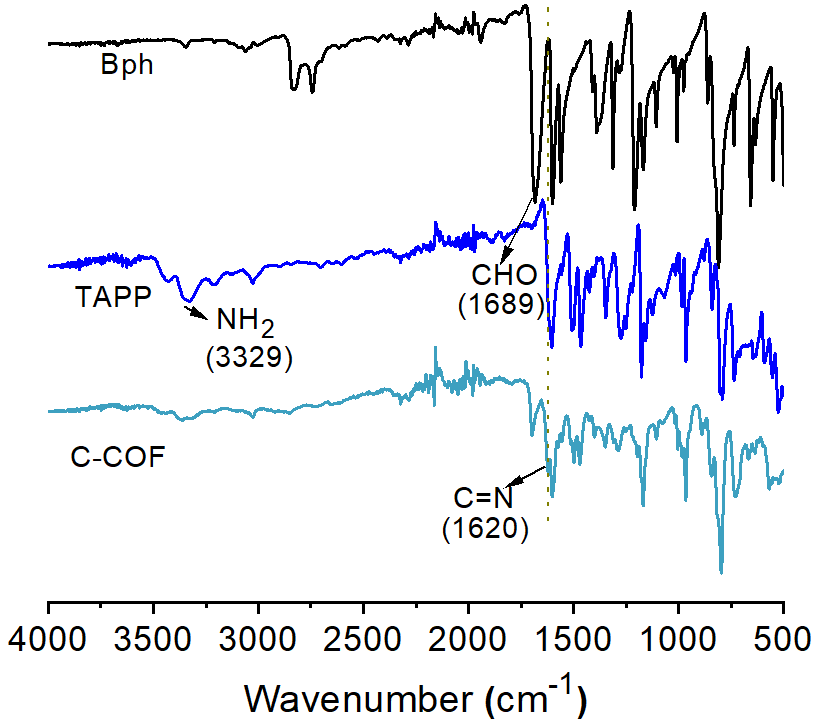
**

**Fig. S8** FT-IR spectra of starting materials of Bph, TAPP and as-prepared C-COF, respectively

**
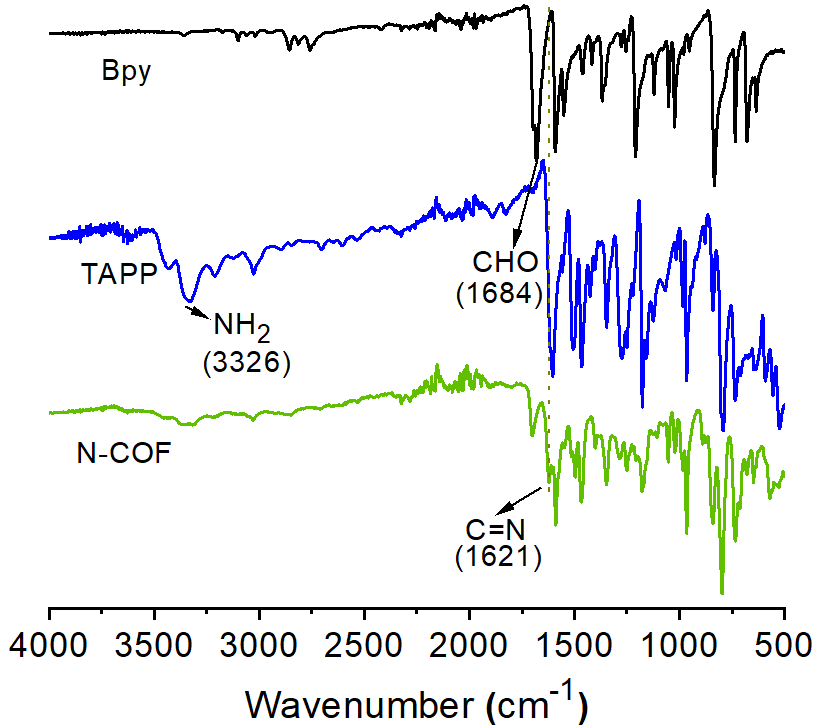
**

**Fig. S9** FT-IR spectra of starting materials of Bpy, TAPP and as-prepared N-COF

**
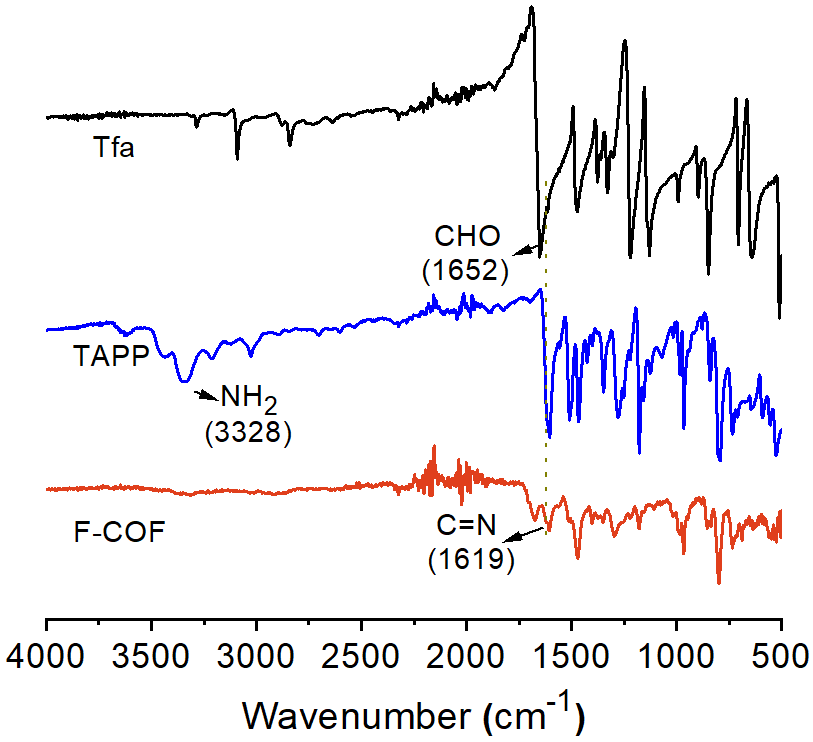
**

**Fig. S10** FT-IR spectra of starting materials of Tfa, TAPP and as-prepared F-COF


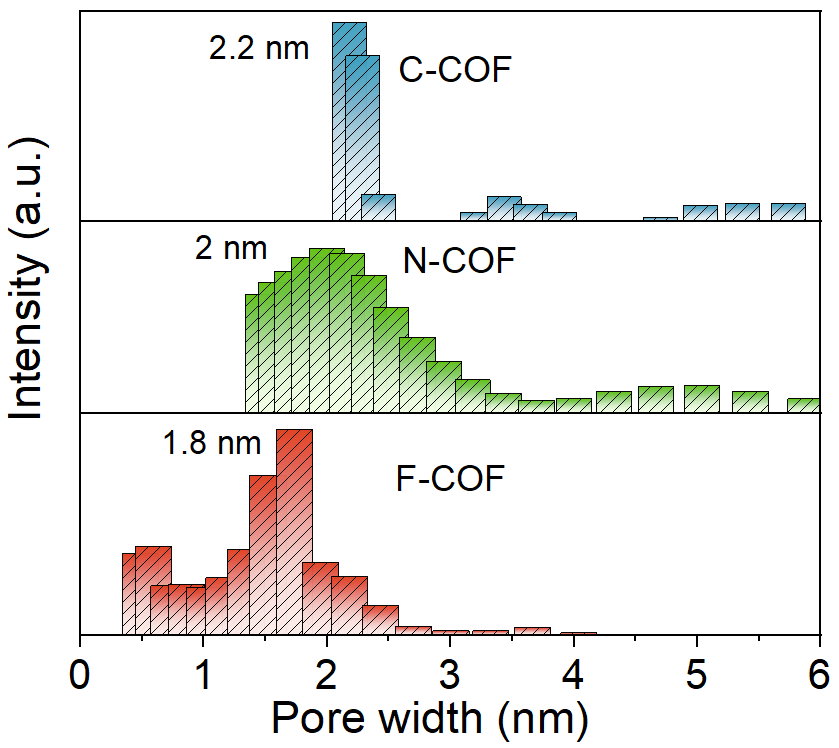


**Fig. S11** Pore size distribution of C-COF, N-COF and F-COF


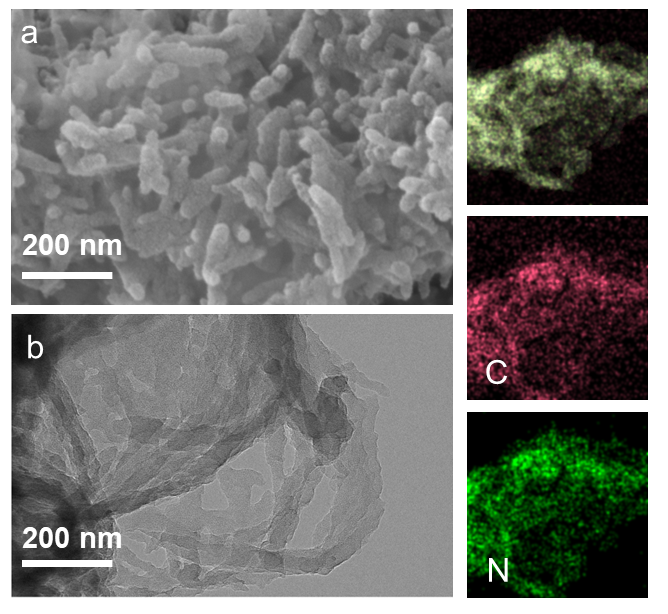


**Fig. S12** SEM (**a**) image of C-COF, TEM (**b**) and EDS mapping C-COF


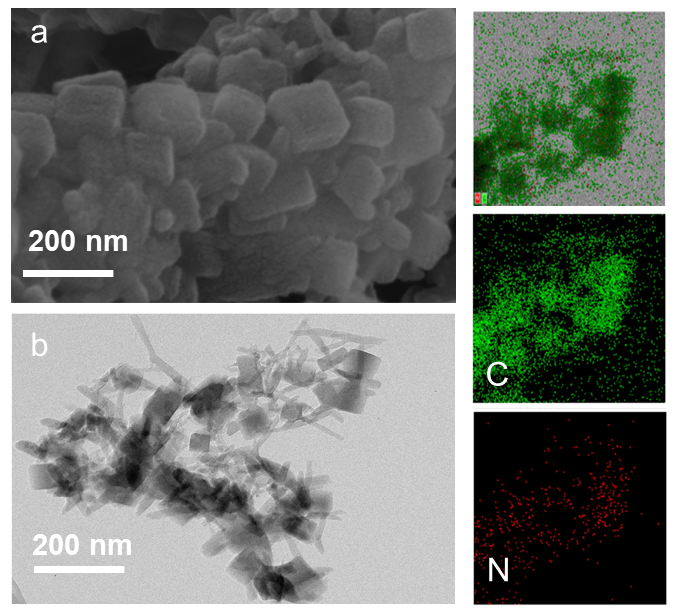


**Fig. S13** SEM (**a**) image of C-COF, TEM (**b**) and EDS mapping N-COF


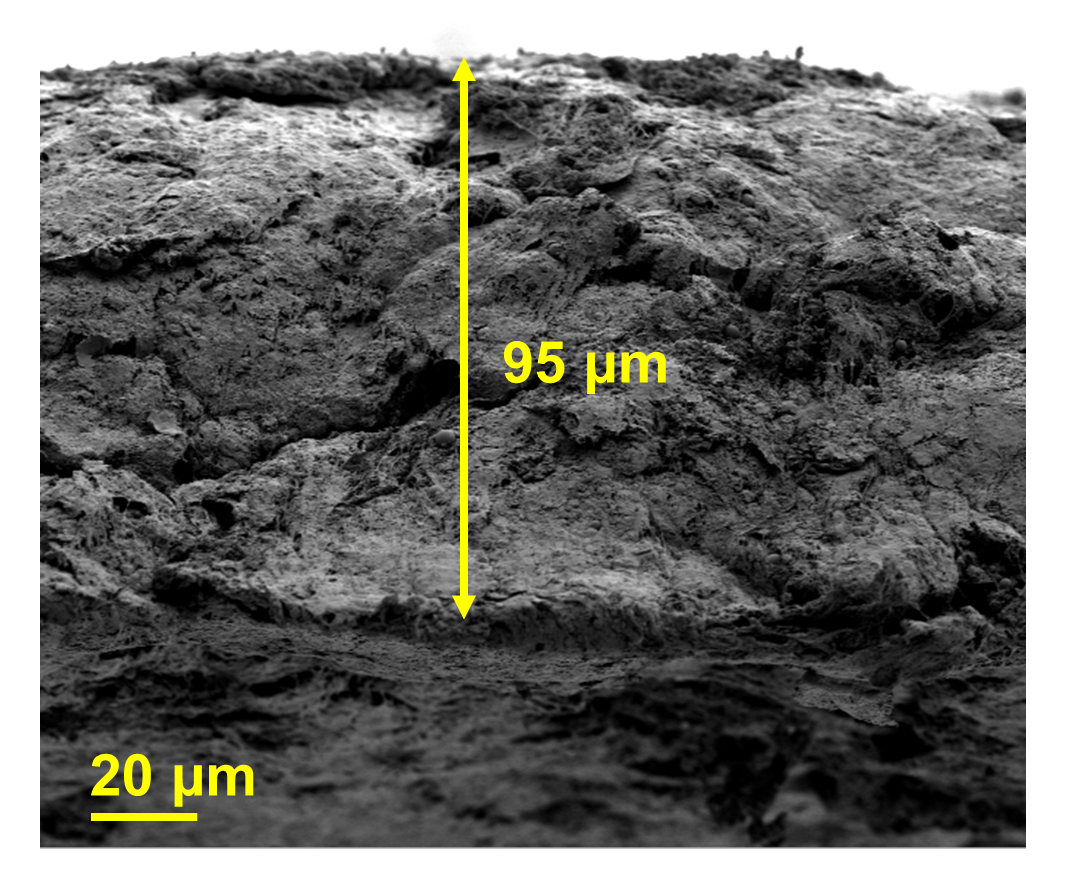


**Fig. S14** Cross-sectional SEM image of F-COF SSE membrane


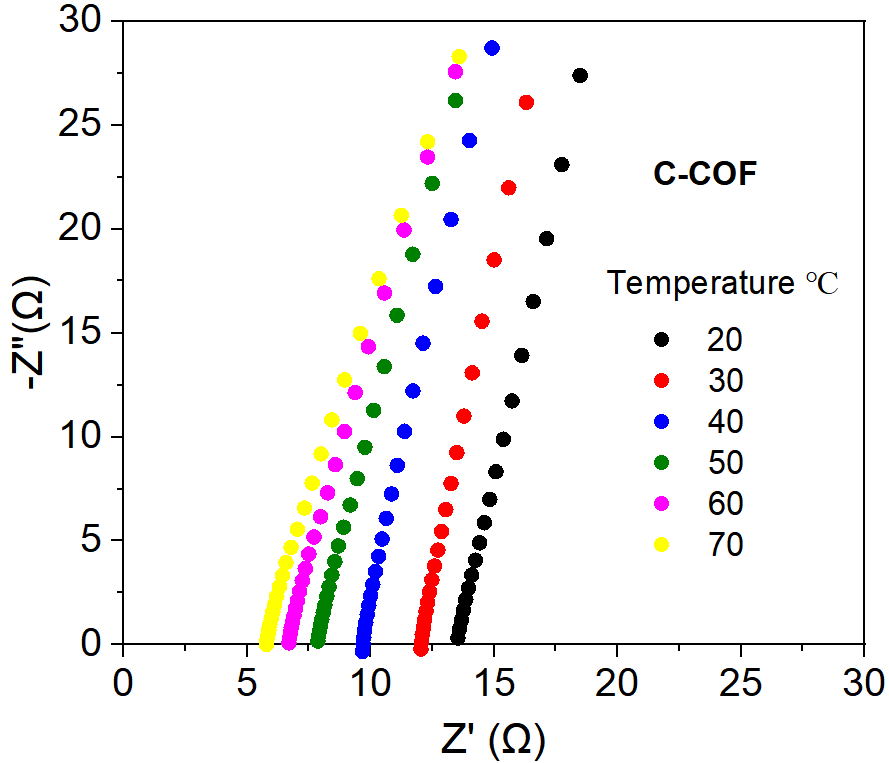


**Fig. S15** EIS spectra of C-COF SSE from 20 to 70 °C


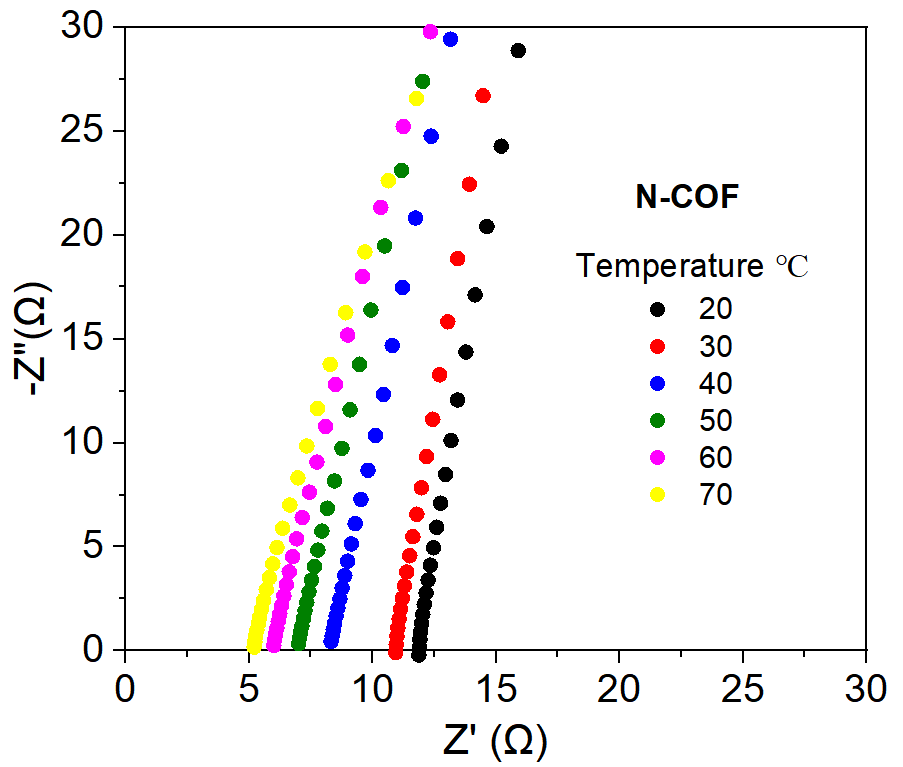


**Fig. S16** EIS spectra of N-COF SSE from 20 to 70 °C


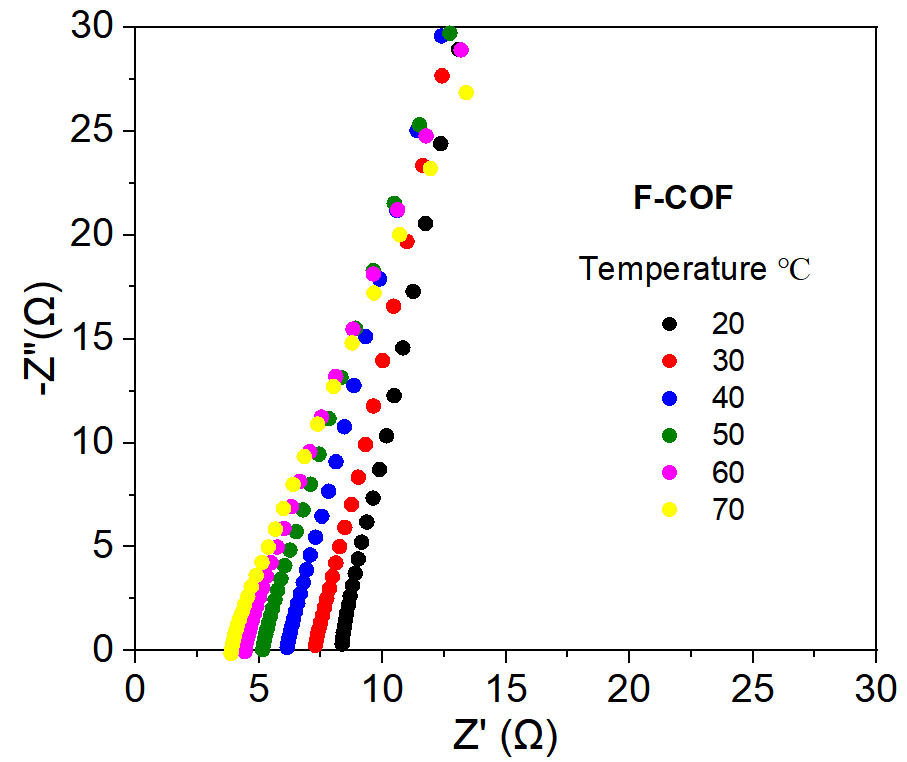


**Fig. S17** EIS spectra of F-COF SSE from 20 to 70 °C


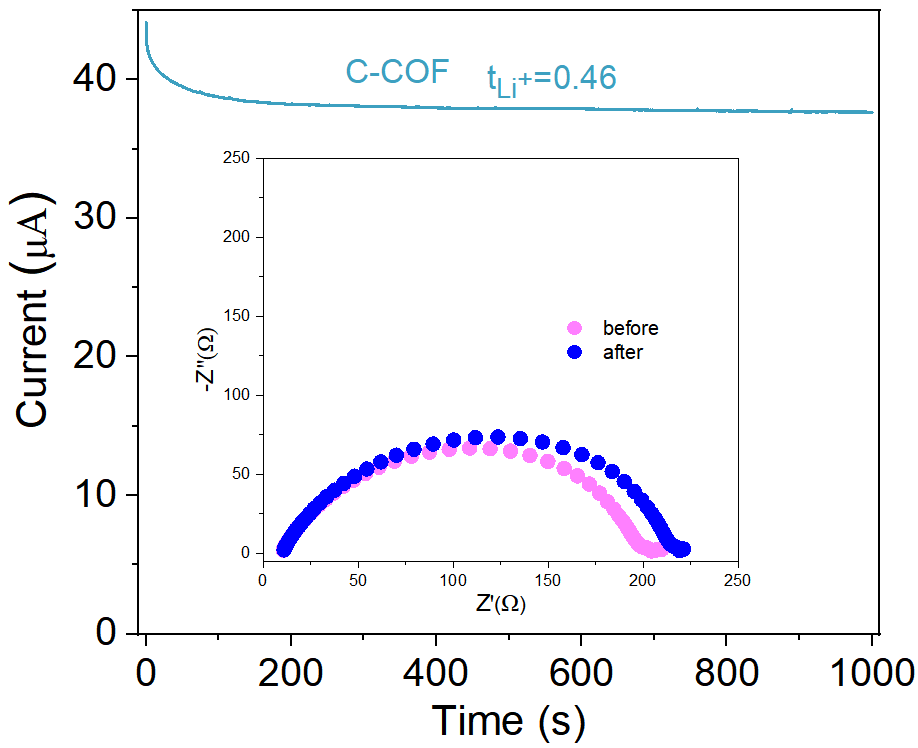


**Fig. S18** Current-time curve of Li|C-COF|Li cell (inset: EIS spectra of Li|C-COF|Li cell before and after polarization


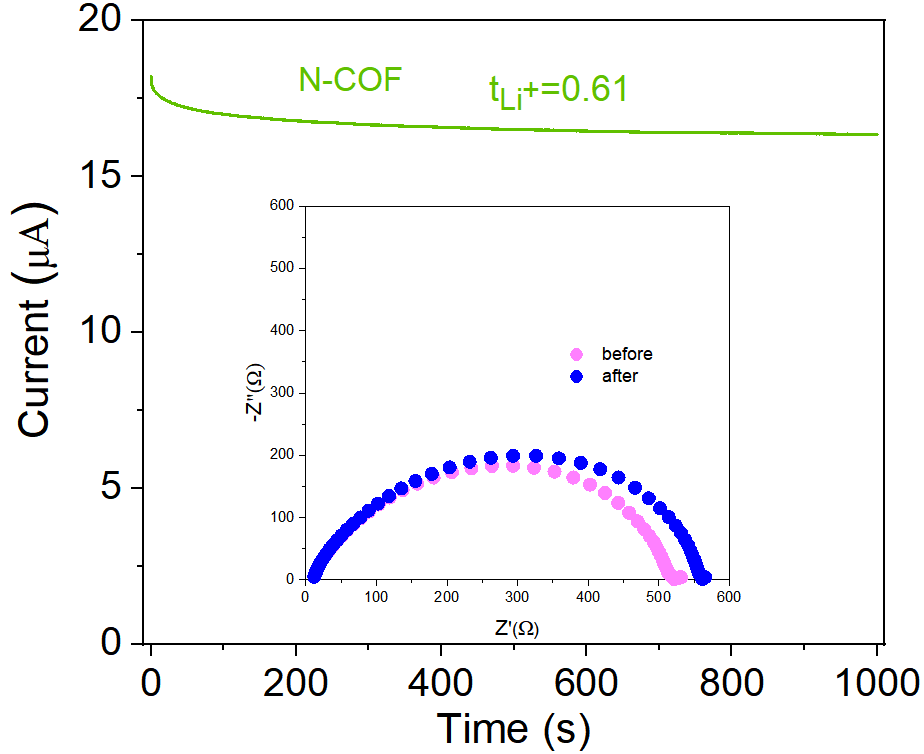


**Fig. S19** Current-time curve of Li|N-COF|Li cell (inset: EIS spectra of Li|N-COF|Li cell before and after polarization


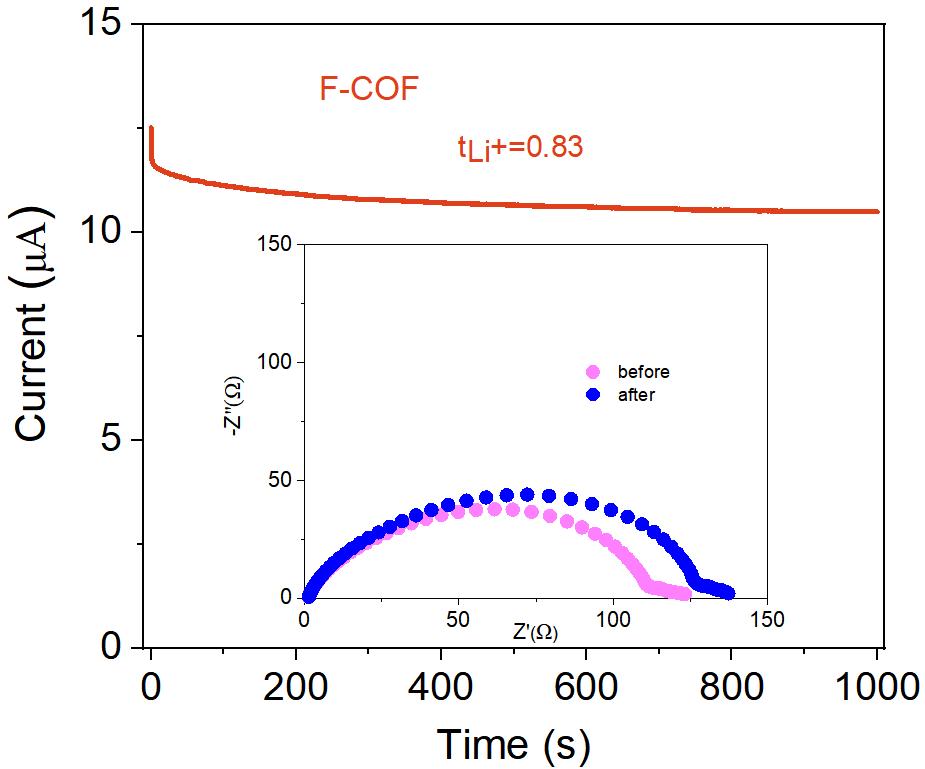


**Fig. S20** Current-time curve of Li|F-COF|Li cell (inset: EIS spectra of Li|F-COF|Li cell before and after polarization


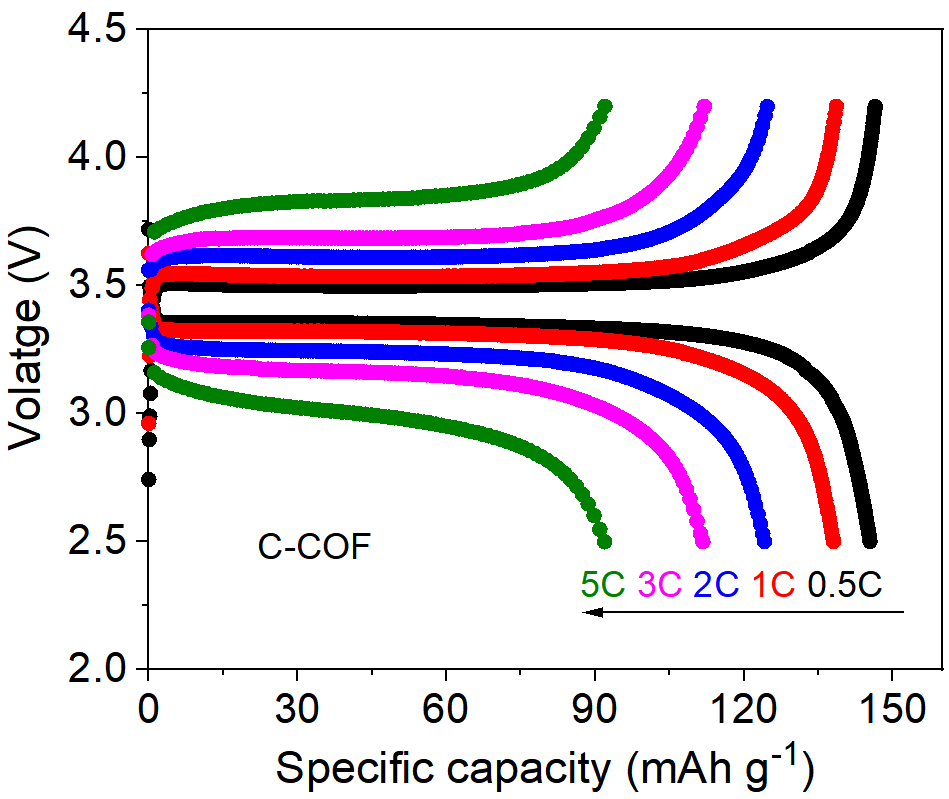


**Fig. S21** The charge-discharge curves of Li|C-COF SSE|LFP quasi-solid-state battery at 0.5, 1, 2, 3 and 5C


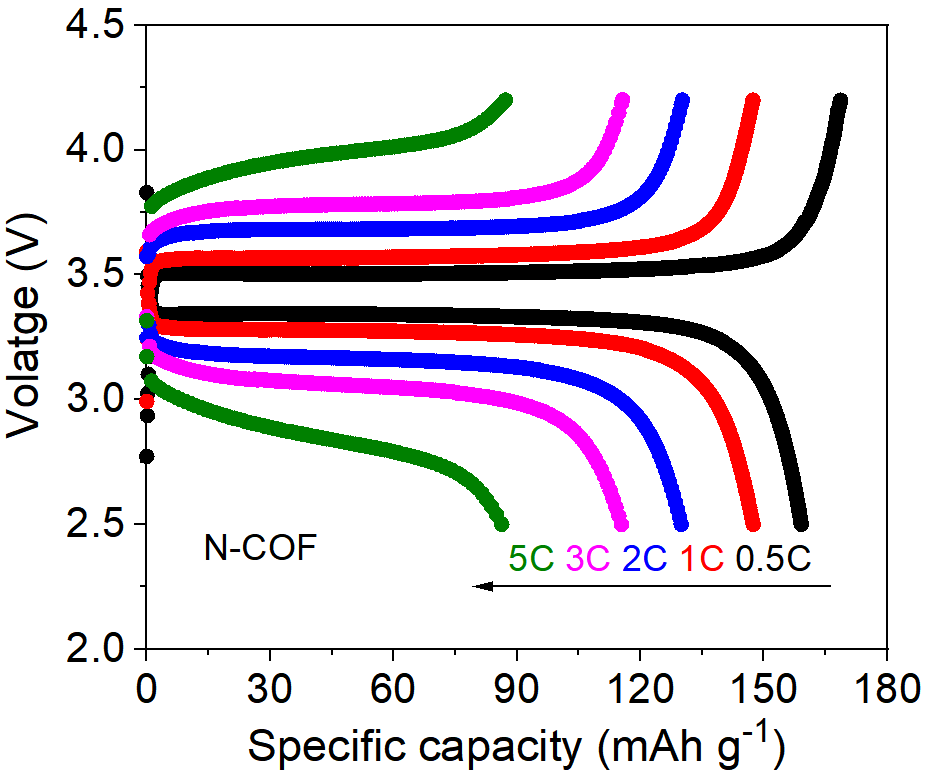


**Fig. S22** The charge-discharge curves of Li|N-COF SSE|LFP quasi-solid-state battery at 0.5, 1, 2, 3 and 5C


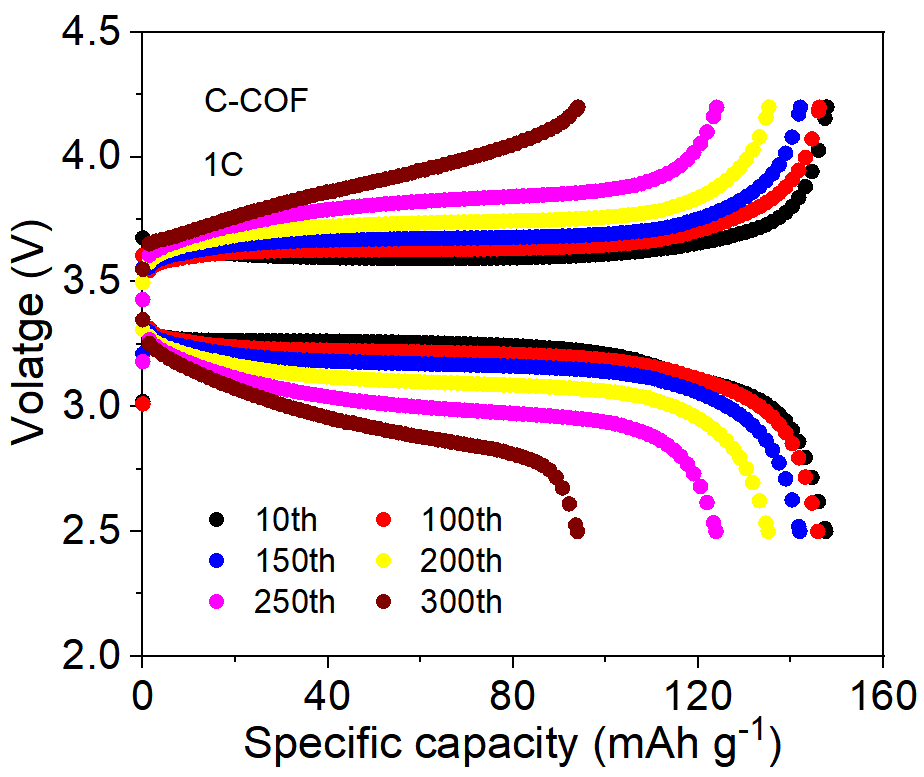


**Fig. S23** The charge-discharge curves of Li|C-COF SSE|LFP quasi-solid-state battery at various cycle


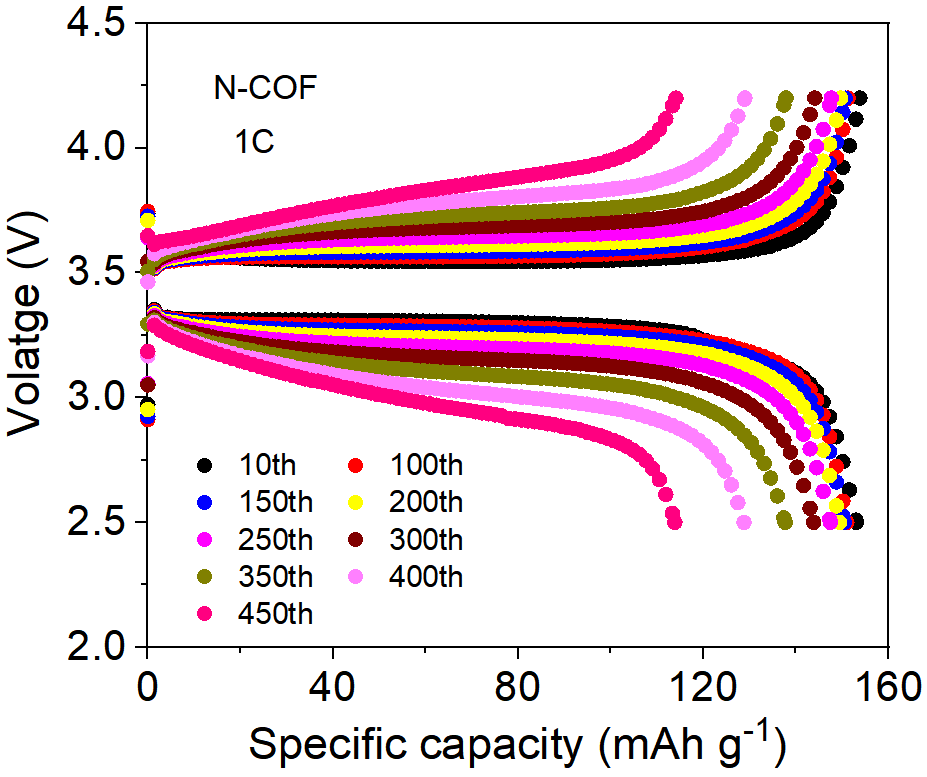


**Fig. S24** The charge-discharge curves of Li|N-COF SSE|LFP quasi-solid-state battery at various cycle

**Supplementary References**

1. G. Kresse, J. Furthmüller, Efficient iterative schemes for ab initio total-energy calculations using a plane-wave basis set. Phys. Rev. B **54**, 11169 (1996). <https://doi.org/10.1103/PhysRevB.54.11169>
2. J. P. Perdew, K. Burke, M. Ernzerhof, Generalized Gradient Approximation Made Simple. Phys. Rev. Lett. **77**, 3865 (1996). <https://doi.org/10.1103/PhysRevLett.77.3865>
3. P. E. Blochl, Phys. Rev. B: Condens. Projector augmented-wave method. Matter Mater. Phys. **50**, 17953−17979 (1994). <https://doi.org/10.1103/PhysRevB.50.17953>
4. H. Jónsson, G. Mills, K. W. Jacobsen, *Nudged elastic band method for finding minimum energy paths of transitions*. Proceedings of the International School of Physics, LERICI, Villa Marigola (1998). <https://doi.org/10.1142/9789812839664_0016>
